# Evaluation of LIGHT-derived peptides to disrupt the HVEM/LIGHT immune checkpoint

**DOI:** 10.1038/s41598-025-23946-4

**Published:** 2025-11-17

**Authors:** Piotr Ciura, Simon Gumpelmair, Sylwia Rodziewicz-Motowidło, Peter Steinberger, Marta Spodzieja, Adam K. Sieradzan

**Affiliations:** 1 Faculty of Chemistry, Department of Theoretical Chemistry, University of Gdańsk, Wita Stwosza 63, Gdańsk, 80-308 Poland; 2 Division of Immune Receptors and T cell Activation, Institute of Immunology, Centre for Pathophysiology, Infectiology and Immunology, Medical University of Vienna, Lazarettgasse 19, Vienna, 1090 Austria; 3 Faculty of Chemistry, Department of Biomedical Chemistry, University of Gdańsk, Wita Stwosza 63, Gdańsk, 80-308 Poland

**Keywords:** Disulfide-linked peptide, ELISA, Cellular assay, Spectral shift, MMGBSA, Computational biology and bioinformatics, Drug discovery, Immunology

## Abstract

Immune checkpoints are critical regulators of immune homeostasis and have become prominent targets in the treatment of various malignancies and autoimmune disorders. While monoclonal antibodies currently dominate checkpoint-targeted therapies, there is a growing interest in alternative approaches, which may offer advantages including smaller size, greater specificity, ease of manufacturing, or a reduced risk of immune-related adverse events. In this study, we focused on the HVEM and its ligand LIGHT, a receptor-ligand pair involved in modulating T cell responses. Overstimulation of HVEM/LIGHT pathway is associated with the pathogenesis of autoimmune diseases and can lead to graft rejection. Therefore, targeting this interaction may offer novel therapeutic strategies for inducing immunosuppression and preventing graft rejection. Utilizing computational approaches, we designed a series of LIGHT-derived peptides. Various simulation techniques, such as molecular dynamics with MMGBSA analyses (with and without NMA), work associated with conformal change and SMD were employed to predict their binding affinities to HVEM. Selected peptides were synthesized and subjected to experimental validation to assess their binding capabilities to HVEM, determined using the spectral shift technique, and inhibitory effects on the HVEM/LIGHT complex formation, evaluated through immunoenzymatic assays and cellular studies. Among these, two peptides demonstrated inhibitory potential, suggesting that they might have utility as scaffold for further optimization. These findings underscore the potential of peptide-based inhibitors in modulating immune checkpoints and pave the way for novel immunotherapeutic strategies.

**Supplementary Information:**

The online version contains supplementary material available at 10.1038/s41598-025-23946-4.

## Introduction

The immune response is precisely regulated through a complex network of co-stimulatory and co-inhibitory signals orchestrated by immune checkpoints (ICPs), which play a crucial role in maintaining immunological balance^1,2^. Herpes virus entry mediator (HVEM) and its ligand – homologous to lymphotoxins, exhibits inducible expression, and competes with HSV glycoprotein D for herpes virus entry mediator, a receptor expressed by T lymphocytes (LIGHT) are proteins belonging to stimulatory checkpoints, and their interaction leads to the stimulation of the immune system, resulting in activation and proliferation of naïve T cells, and the secretion of pro-inflammatory cytokines. The immune system utilizes this complex to combat threats, but its dysregulation can contribute to immune pathologies, making it a valuable target for therapeutic intervention^1–3^. Previous studies have shown that blockade of the HVEM/LIGHT interaction with the use of antibodies or soluble receptors can reduce allogeneic immune responses, improve graft survival, and mitigate graft-versus-host disease (GVHD)^4,5^. Nevertheless, a need for novel compounds with different pharmacokinetic properties capable of disrupting HVEM/LIGHT interaction and inducing effective immunosuppression remains.

LIGHT is a ligand of the tumour necrosis factor superfamily (TNFSF). The protein is a cell-surface homotrimer. Each monomer is made up of 240 amino acids and divided into three domains: the cytoplasmic (residues 1–37), the transmembrane (residues 38–58), and the extracellular (residues 59–240)^6^. The monomer of the LIGHT is stabilized by one disulfide bond between cysteines at positions 154 and 187. A distinctive feature of LIGHT is the presence of a TNF homology domain (THD) at the *C*-terminal part of the ectodomain, which enables the protein trimerization and is responsible for the interaction of LIGHT with its receptors^5^. This protein can also exist in a soluble form generated through alternative mRNA splicing or proteolytic cleavage of its extracellular domain^7^. The LIGHT interacts with several receptors from TNFSF: the previously mentioned HVEM, decoy receptor 3 (DcR3)^8^ and lymphotoxin β receptor (LTβR)^9^. The binding of the LIGHT with the HVEM and LTβR results in the activation of signalling pathways responsible for the stimulation of the immune system^10^, while its interaction with the DcR3 inhibits immune activation via LIGHT by preventing the formation of the HVEM/LIGHT and LTβR/LIGHT complexes^11^. LIGHT is expressed on dendritic cells (DCs), monocytes, T cells, B cells, and myeloid cells^3^. Its expression on immature DCs implies that LIGHT may have a significant role in the DC-mediated co-stimulation of T cells^5^.

HVEM belongs to the TNFSF receptor, and it is a 283 amino acid protein composed of a signal peptide (residues 1–38), extracellular (39–202), transmembrane (203–223), and intracellular (224–283) parts. The extracellular region of HVEM contains four cysteine-rich domains (CRDs) linked by disulfide bonds^12–14^. Structural information is well-defined for the first three CRDs of HVEM, which are involved in binding receptors from the immunoglobulin superfamily (IgSF), such as B and T-lymphocyte attenuator (BTLA)^15,16^ and cluster of differentiation 160 (CD160)^17^, as well as ligands from TNFSF, such as LIGHT and lymphotoxin α (LTα)^18,19^. HVEM exhibits bifunctional immunomodulatory properties. It can engage with inhibitory receptors such as BTLA and CD160 to suppress T cell activation or function as a receptor for LIGHT and LTα, thereby delivering co-stimulatory signals that enhance T cell activation^20,21^. HVEM is expressed on T cells, B cells, natural killer (NK) cells, DCs, and myeloid cells^13,22^.

The HVEM/LIGHT complex has a 3:3 stoichiometry, in which a trimer of LIGHT binds three molecules of HVEM. CRD2 and CRD3 of HVEM are mainly involved in specific interactions with LIGHT; however, some residues from CRD1 are also engaged in protein binding. The crystal structure of the HVEM/LIGHT complex (PDB code 4RSU) shows that Y173 from DE loop, G100 from AA’ loop, and R226, R228 from GH loop of LIGHT interact with CRD1 and CRD2 of HVEM, while G151, V152, A159–T161 from CD loop, and Q183, R195, V196 and W198 from EF loop of LIGHT bind to CRD3 of HVEM^23^. The interaction of HVEM present on T cells and LIGHT on immature DCs mediates the co-stimulation of T cells, resulting in the activation of several signalling pathways, including nuclear factor kappa-light-chain-enhancer of activated B cells (NF-κB), which is crucial for regulating immune responses, cell survival, and inflammation^24^. Under certain conditions, such as autoimmune diseases characterized by excessive immune activation or in transplant patients it is necessary to dampen immune responses. One promising therapeutic approach involves inhibiting the formation of the HVEM/LIGHT complex, thereby attenuating downstream signalling pathways involved in immune activation. Many studies confirmed that blocking the interaction of LIGHT with its receptors HVEM and LTβR using monoclonal antibodies or soluble form of receptors (LTβR-Ig and HVEM-Ig) reduced allogeneic T cell immune responses in vitro and in vivo^25–27^.

Our research is one of the first to focus on the design of peptide inhibitors based on the LIGHT protein. Agostino et al. also performed the computational analysis and designed number of peptides based on LIGHT protein-binding fragments to HVEM and LTβR, where they showed that those peptides have a potential as therapeutics for the metabolic syndrome, but their affinity to receptors and mechanism of action were not investigated^28^. Our research focused on designing peptides to block HVEM/LIGHT interactions. The crystal structure of the HVEM/LIGHT complex was used as a template to design five compounds for which molecular dynamics (MD) and molecular mechanics generalised Born and surface area continuum solvation (MMGBSA) analyses were performed. The amino acid sequences of these peptides were based on two fragments of LIGHT, the first covering the residues from 210 to 240 (peptides light1 and light2) and the second from 171 to 195 (peptides light3, light4, and light5). The binding of compounds to LIGHT was, at first studied, using computational methods – MMGBSA and steered molecular dynamics (SMD). The highest theoretical affinity and the most stable complex with HVEM was created by light3, and therefore nine analogues of this peptide were designed. In the next step, the selected peptides were studied in more detail: enthalpy, entropy, binding free energy, internal work, total work, and force max for the peptide/HVEM complexes were determined. These peptides were also synthesized and their affinity for the HVEM by spectral shift (SpS) technique, and their inhibition properties by enzyme-linked immunosorbent assay (ELISA) and cellular assays were evaluated. We designed overall 14 peptides based on LIGHT structure, which were termed with lower case “light” and number and mutation (if occuring). The best results were obtained for peptide light3 and its analogue light3_E178L. These compounds are promising candidates for further exploration as immunomodulatory agents.

## Results and discussion

### Peptide design based on various computational approaches

The first step of this study was to design peptides – fragments of the LIGHT protein that can bind to HVEM and thus inhibit the formation of the HVEM/LIGHT complex. For this purpose, MMGBSA analysis performed for the crystal structure of the HVEM/LIGHT complex (PDB code 4RSU, chains A-D) were used to determine key amino acids within the LIGHT that are responsible for binding to HVEM (Figure S1). The analysis was carried out for a complex consisting of a LIGHT trimer and a HVEM monomer. This method allows for a relatively quick and low-cost estimation of the energy of the interaction between the target and the ligand^29,30^. Per-residue and pairwise per-residue energy decomposition were also performed to determine the interaction energy of individual residues and their contribution to the interaction with HVEM.

**Fig. 1:**
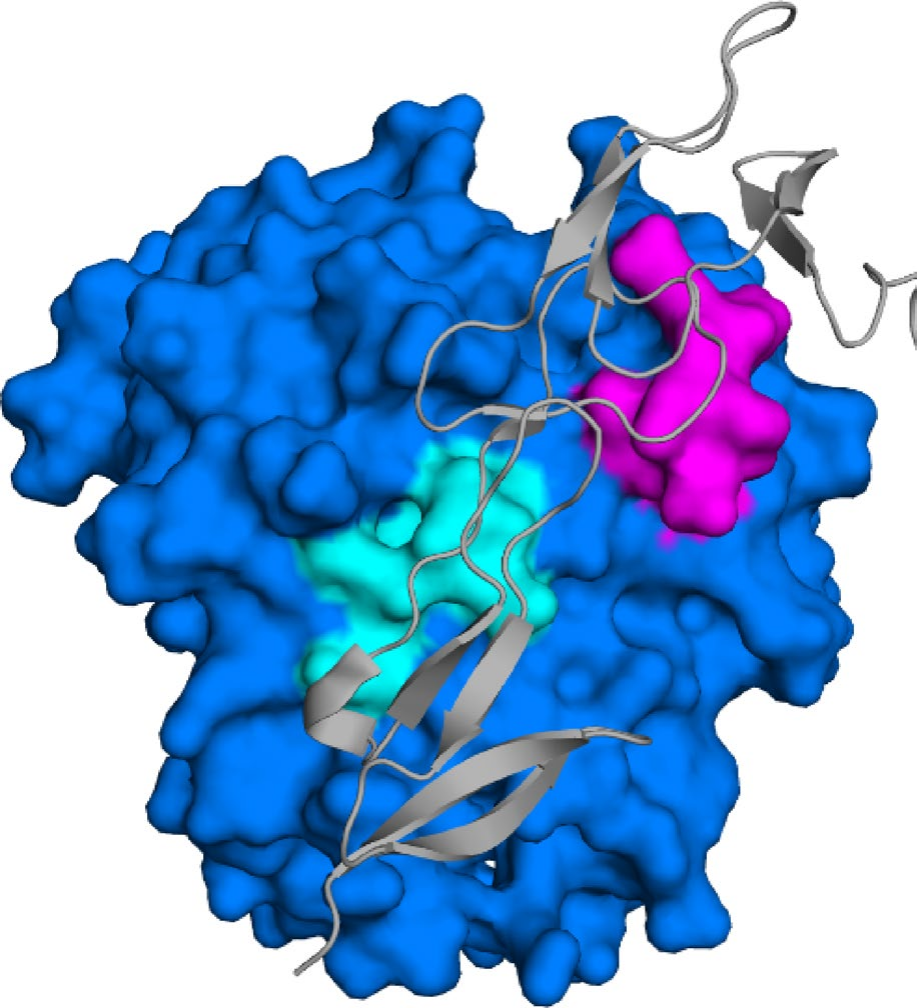
Structure of the HVEM (grey ribbon)/LIGHT (blue surface) complex with two binding regions of the LIGHT highlighted: P171–L177 (magenta) used for the design of light3, light4, and light5 peptides, and R226–F235 (cyan), used for the development of light1 and light2.

Analyses of the HVEM/LIGHT complex, carried out, revealed that HVEM interacts with two monomers of LIGHT, binding to residues 171–177 of the first LIGHT molecule and five regions: T99–L105, E115–F122, Q148–V152, S192–W198 and R226–F235, of the second LIGHT molecule. Per-residue and pairwise per-residue energy decomposition indicate that the lowest energy values were observed for the fragment R226–R228 and residue R172 in LIGHT, confirming that these amino acids are essential for interaction with the receptor (Figure S1). The crystal structure of the HVEM/LIGHT complex, including the binding sites that served as the basis for peptide design, is shown in Fig. 1. Based on these results, five peptides were initially designated namely: light1 and light2, covering the fragment 210–240 of LIGHT, and light3, light4, and light5, based on the residues of 161–185 of LIGHT.

The fragments of LIGHT involved in the interaction with HVEM adopt a β-harpin conformation. To stabilize the structure of the designed peptides the appropriate amino acid sequences were elongated and disulfide bonds were introduced by substituting selected residues with cysteines. This strategy, previously employed by us and others, has demonstrated that both the presence and the position of a disulfide bond within a peptide can be critical for its interaction with a molecular target^31–36^. The amino acid sequences of the peptides are shown in Table 1 (Peptide 1–5).

### Short molecular dynamics simulations and their analyses reveal most promissing inhibitors

**Fig. 2:**
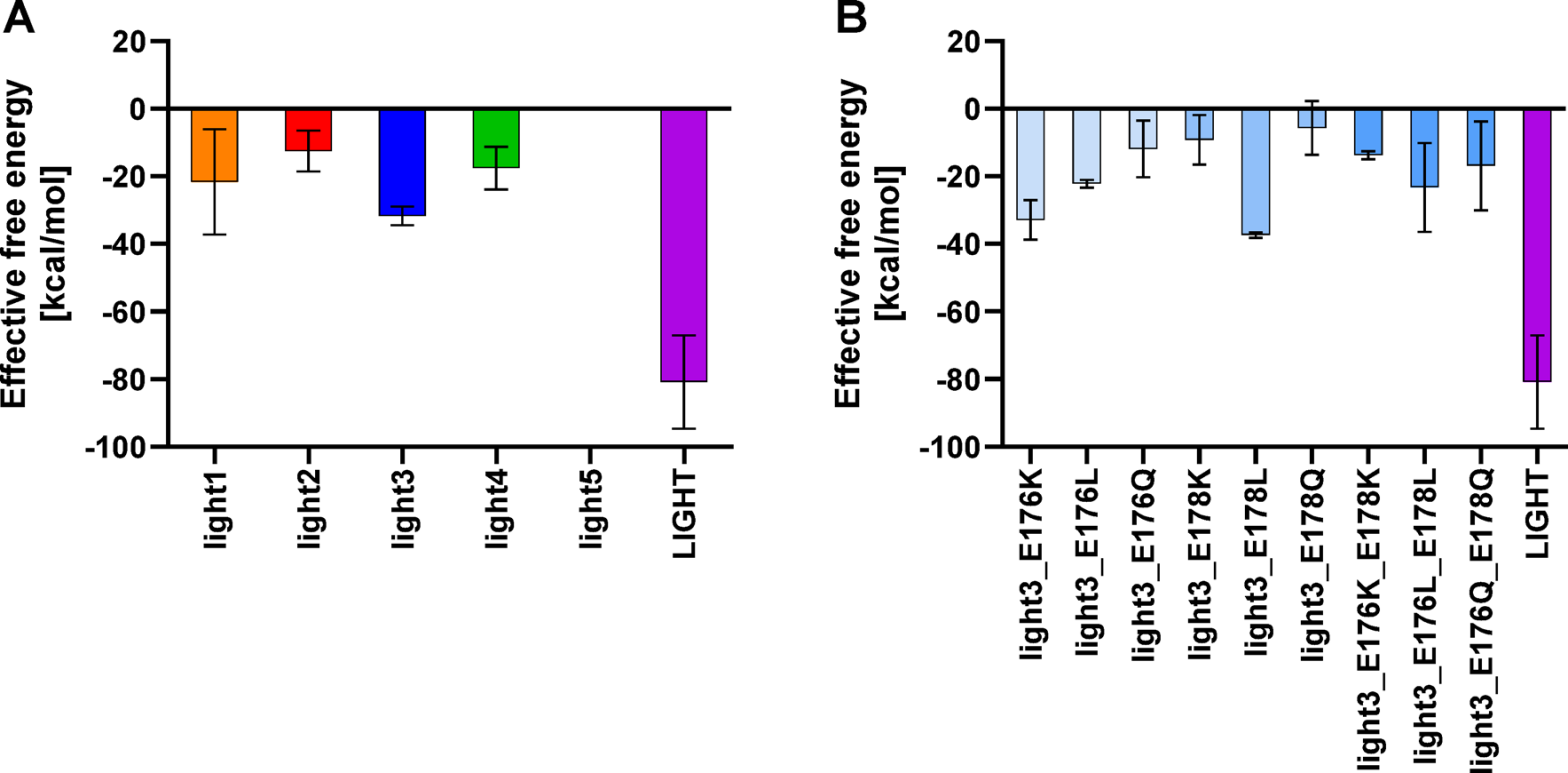
Diagram of MMGBSA – derived effective binding free energy for 200 ns simulations for initial peptides (**A**) and light3 peptide analogues (**B**). The results are presented as mean ± SD based on analyses from three independent trajectories.

Next, short molecular dynamics simulation (200 ns) were conducted to estimate the binding affinity of all of the designed peptides to HVEM. The RMSD analysis confirmed that peptides reach equilibrium within 200 ns (Figure S2). In addition, longer simulations (1000 ns) were carried out for a subset of selected complexes to gain deeper insights into the nature of the peptide/HVEM interactions. Based on results obtained from short simulations, light5 was excluded from future studies, because it did not form a stable complex with receptor (Fig. 2A) whereas light2 and light4 exhibited very weak binding to HVEM. A similar pattern was observed in case of long simulations (Figure S3). The strongest interaction with the HVEM was obtained for the light3 (Fig. 2A and S3); therefore, the effective theoretical binding energy decomposition (per-residue and pairwise per-residue) for the light3/HVEM complex was performed to identify key amino acids contributing to binding with HVEM.

### Determination of substitution directions to improve affinity based on per-residue and pairwise decomposition

**Fig. 3:**
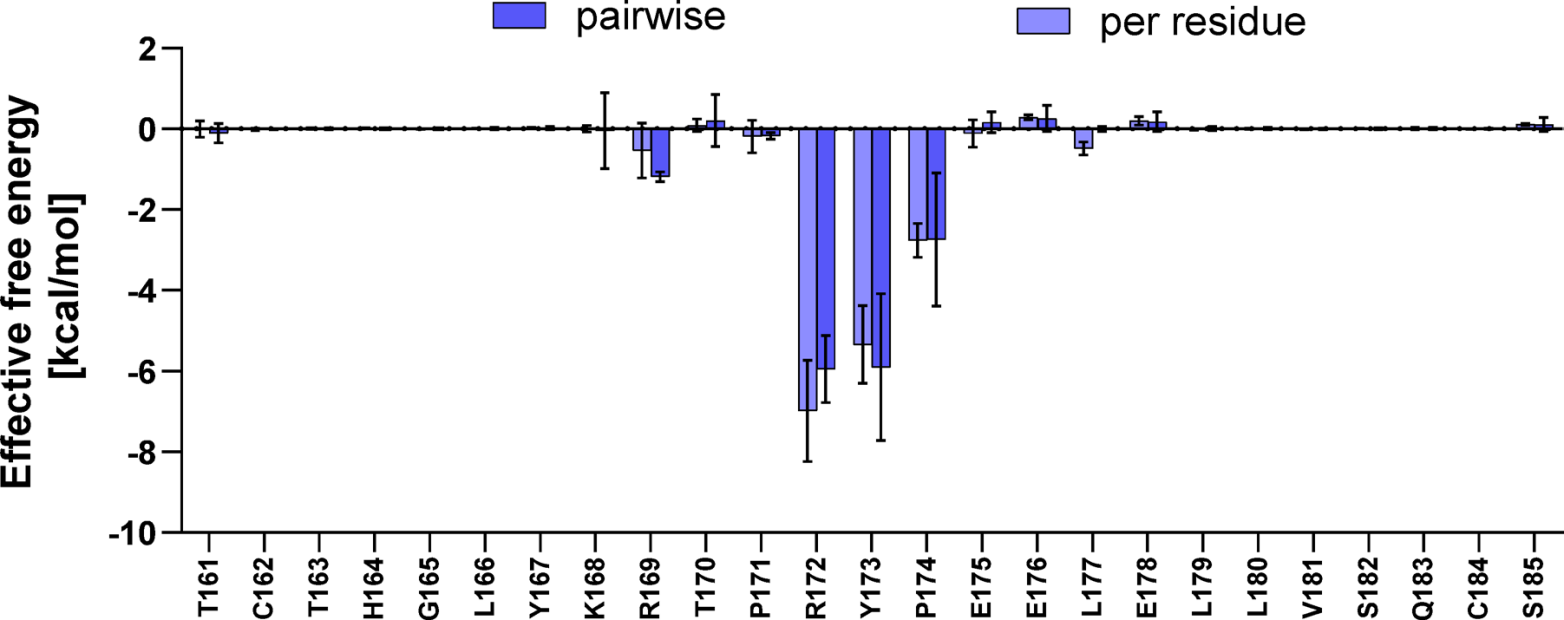
MMGBSA effective theoretical binding energy decomposition (per-residue and pairwise) calculated for light3 peptide. Results are shown for 3 independent trajectories as mean ± SD.

The energy values obtained from both, per-residue and pairwise per-residue decomposition indicated that R172, Y173, and P174 in light3 are crucial for the interaction with HVEM, whereas the remaining residues in the peptide have no significant effect on the binding to the protein. This was also confirmed by LigPlot +^37^ analysis (Figure S4). The spatial analysis of representative structure of light3 reveals that those residues bind to HVEM-formed grove. This grove is formed by residues L1, Y9–V11 and I71–L77 from HVEM (Figure S5). A potentially minor destabilizing effect of amino acids E176 and E178 on the protein/peptide complex was observed (Fig. 3). Therefore, in the next step, these residues were substituted by lysine, leucine and glutamine (Table 1, Peptides 6–16). The negatively charged Glu178 of Light3, in conjunction with Asp97 and Asp99 of HVEM, electrostatically disfavors the formation of an extended interaction surface (Figure S6). These amino acid substitutions were chosen to change the chemical nature of these residues – aimed to alter the properties associated with the side chain of glutamic acid – opposite charge, hydrophobic residue with similar size and neutral charge with the same size, respectively, were selected). Structural analysis of light3 allowed to conclude that these residues are not located in close proximity to the HVEM protein and therefore do not have an impact on interaction with HVEM. Rather they seem to have a destabilizing effect on the peptide itself as both negatively charged residues are in close proximity. Their electrostatic repulsion may contribute to an unfavourable entropic effect on the peptide structure; therefore, substituting these residues could potentially enhance the peptide/HVEM interactions. The best result was obtained for the peptide light3_E178L, in which glutamic acid in position 178 was replaced by leucine (Fig. 2B).

**Table 1:** The amino acid sequence of the designed peptides, fragments of LIGHT protein; & – position of disulfide bond. Methionine in synthesized peptides has been replaced with norleucine.

No.	Peptide designation	Amino acid sequence
1	light1	Ac-E^210^AGEEVVC(&)RVL^220^DERLVRLRDG^230^TRSYC(&)GAFMV^240^-NH_2_
2	light2	Ac-E^210^C(&)GEEVVVRVL^220^DERLVRLRDG^230^TRSYFGAFC(&)V^240^-NH_2_
3	light3	Ac-TC(&)THGLYKRT^170^PRYPEELELL^180^VSQC(&)S-NH_2_
4	light4	Ac-TIC(&)HGLYKRT^170^PRYPEELELL^180^VSC(&)QS-NH_2_
5	light5	Ac-TITC(&)GLYKRT^170^PRYPEELELL^180^VC(&)QQS-NH_2_
6	light3_E176K	Ac-TC(&)THGLYKRT^170^PRYPEKLELL^180^VSQC(&)S-NH_2_
7	light3_E176L	Ac-TC(&)THGLYKRT^170^PRYPELLELL^180^VSQC(&)S-NH_2_
8	light3_E176Q	Ac-TC(&)THGLYKRT^170^PRYPEQLELL^180^VSQC(&)S-NH_2_
9	light3_E178K	Ac-TC(&)THGLYKRT^170^PRYPEELKLL^180^VSQC(&)S-NH_2_
10	light3_E178L	Ac-TC(&)THGLYKRT^170^PRYPEELLLL^180^VSQC(&)S-NH_2_
11	light3_E178Q	Ac-TC(&)THGLYKRT^170^PRYPEELQLL^180^VSQC(&)S-NH_2_
12	light3_E176K_E178K	Ac-TC(&)THGLYKRT^170^PRYPEKLKLL^180^VSQC(&)S-NH_2_
13	light3_E176L_E178L	Ac-TC(&)THGLYKRT^170^PRYPELLLLL^180^VSQC(&)S-NH_2_
14	light3_E176Q_E178Q	Ac-TC(&)THGLYKRT^170^PRYPEQLQLL^180^VSQC(&)S-NH_2_

It should be noted that regions of LIGHT established in our work are similar to previously established by Agostino et al^28^. Their computational analysis lead to designing of several peptides based on LIGHT fragments that interact with HVEM and LTβR, covering residues 98–117, 166–180, and 19–22 ^28, 29^, . Their in vitro studies indicated that peptide 2 composed of the amino acid from 166 to 180 promotes insulin sensitivity and its sequence was optimized using computational methods. The authors developed six analogues of this peptide, each containing a disulfide bond to stabilize the β-hairpin conformation (similarly as in our case) and conducted a series of in vitro and in vivo experiments. Their results suggest that peptides derived from LIGHT could be a novel class of therapeutics for obesity and type 2 diabetes by improving glucose and lipid metabolism.

In our previous studies, using molecular dynamics simulations and MMGBSA analysis, we designed several peptides covering the CRD2 and CRD3 of the HVEM, responsible for interacting with LIGHT. Next we demonstrated that two peptides (CRD2(39–73) and CRD2_K54E) have the highest inhibitory potential to disrupt HVEM/LIGHT complex formation in molecular dynamics simulations^38^, which was also confirmed by experimental studies (Ciura et al. submitted). Taken together, these results highlight the utility of computational methods in both the design and affinity prediction of target-specific compounds.

### The study of peptide/HVEM binding using SMD

As MMGBSA does not always reveal all important aspects of binding properties^38^, therefore, for subsequent analyses, peptides light1–4 and the most promising of light3 analogue, light3_E178L, were selected for SMD simulations. This method, which involves the application of a mechanical force to gradually displace the peptide from the binding site on HVEM, provides valuable quantitative data regarding the strength and stability of peptide/protein interactions. The binding strength was evaluated based on the force required for unbinding and the work performed during the pulling process^39,40^.

**Fig. 4:**
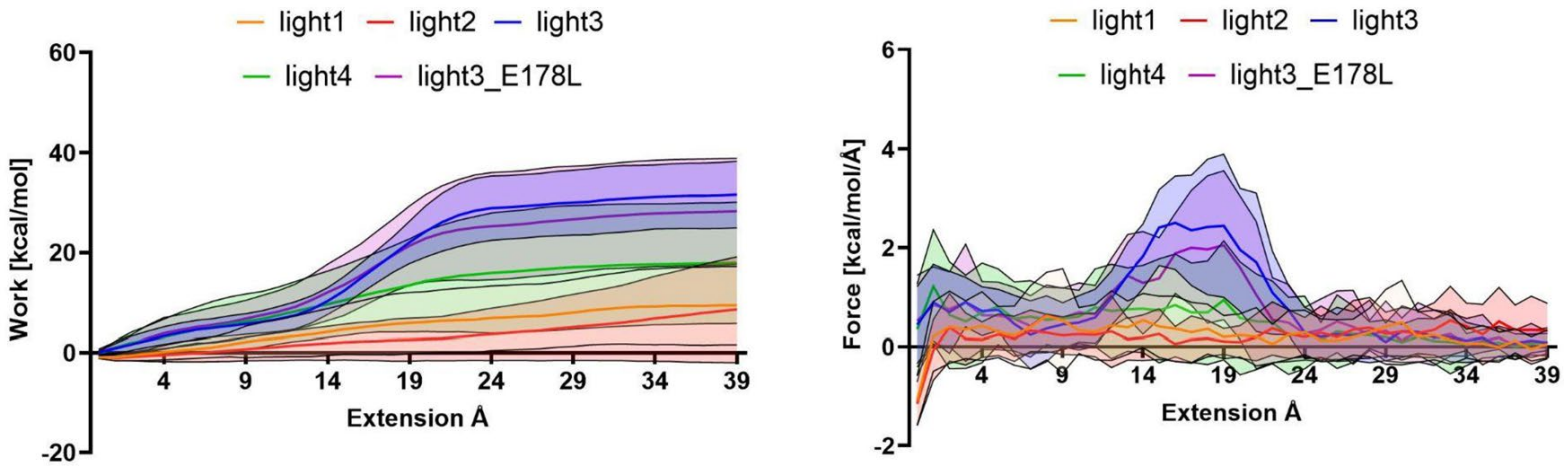
Diagram of total work (**A**) and maximum force (**B**) necessary for dissociation of the peptide/HVEM complex. The results are presented as mean ± SD.

Among the tested peptides, light3 and light3_E178L required the highest work to dissociate from the HVEM protein, indicating the strongest theoretical binding affinity (Fig. 4A). Additionally, detachment of light3 and light3_E178L, is associated with significant energy barriers, which is revealed as a pronounced force peak at around 19Å between centres of mass (Fig. 4B). In contrast, no such force spikes were observed for the other peptides, whose dissociation occurred in a more gradual and unresisted manner, indicative of weaker interactions with the molecular target (Fig. 4A and B). These findings further support the conclusion that light3 and light3_E178L exhibit the highest theoretical binding affinity toward HVEM and are in correlation with previous findings from both long and short MD simulations.

**Fig. 5:**
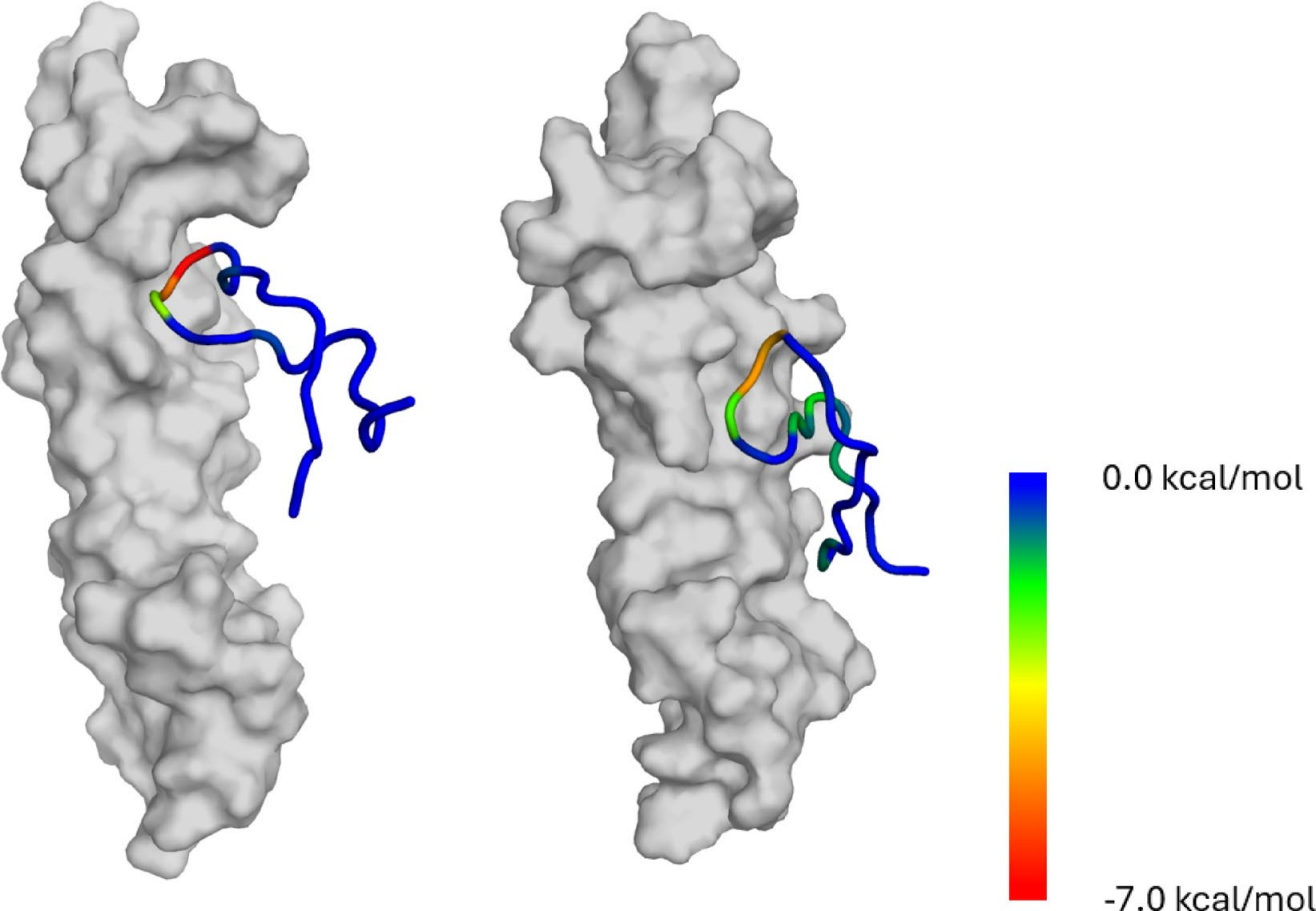
Representative models of light3 (left) and light3_E178L (right), obtained by clustering three independent trajectories, coloured by the per-residue effective theoretical binding energy. The influence of each residue to total binding energy was marked as color in rainbow style ranging from 0 (blue) to −7.0 kcal/mol (red), with HVEM protein presented as grey surface.

Representative structures of the light3 and light3_E178L peptides were obtained by clustering the conformations at extension 19 ± 0.5Å, corresponding to the largest force and work peaks identified in the SMD simulations (Fig. 5). Structural analysis of these, revealed notable differences in the interaction interfaces between the light3/HVEM and its analogue, light3_E178L/HVEM. The light3 peptide engages the HVEM receptor through a localized interaction hotspot, primarily involving residues R172 and Y173. In contrast, the light3_E178L establishes contacts with a broader set of HVEM residues; in the analysis of the starting structure interactions in SMD appear to be more dispersed and potentially weaker (Fig. 5), suggesting a different binding mode. Similar properties were noted in our earlier work when the shortening of the peptide, modified to improve affinity for LIGHT, also altered the character of the interaction plane^38^.

### Analysis of changes in thermodynamic values of peptides in complex with HVEM

**Fig. 6:**
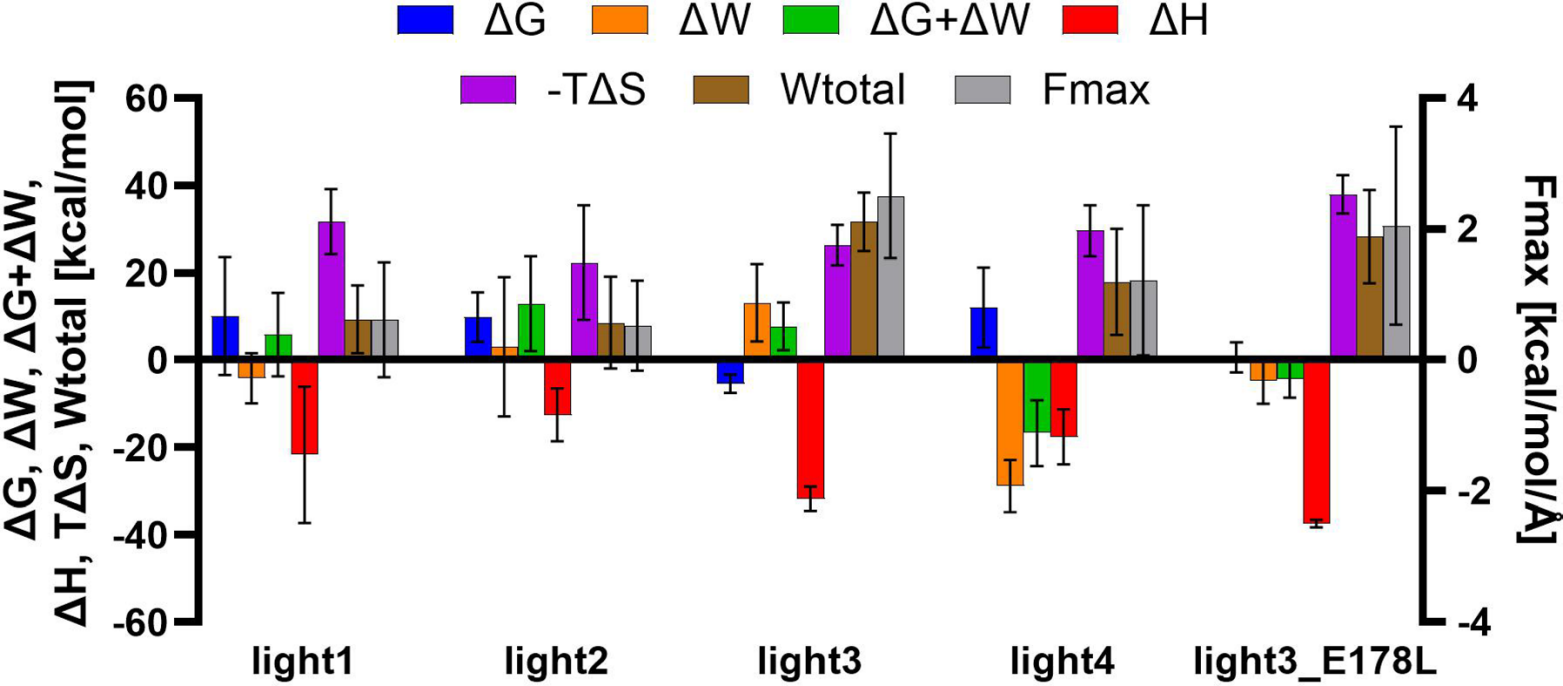
Diagram presenting change of: Gibbs free energy, internal work, sum of Gibbs free energy and internal work, free enthalpy, and entropy for the four determined for peptides and the light3 peptide analogue.

To gain deeper insights into the thermodynamic properties of the peptide/HVEM complexes and allow for the future sequence optimisation, we analysed several energetic parameters such as Gibbs free energy (ΔG), internal work (ΔW), the sum of these two values (ΔG + ΔW), free enthalpy of complex formation (ΔH), and entropy (TΔS). In addition, we also analyzed the values ​​of Fmax (maximum force determined during dissociation) and Wtotal (work determined at maximum dissociation). Together these values allow for a deeper understanding of the nature of the peptide/protein interaction, which was proven in our previous work^38^. The results of these analyses were summarised in Fig. 6. The entropic contribution (TΔS, magenta) reveals the unfavourable effect of reduced conformational freedom following binding. These parameters were analysed for both short and, for comparison, long (Figure S3) simulation timescales. Wtotal and Fmax obtained from SMD simulations were also included in the Fig. 6 to provide a deeper understanding of the interactions between peptides and the HVEM and to achieve a more comprehensive analysis. Similar analyses in our previous study allowed for detailed analyses of peptide/protein interactions and confirmed peptide affinity for the molecular target, as determined by independent methods^38^.

The analyses indicate that for peptides light1, light2 and light4, the TΔS contribution dominates over the ΔH, resulting in positive ΔG value, which suggest a low affinity of these peptides to the molecular target. In contrast, peptides light3 and light3_E178L exhibit a dominant or similar ΔH contribution over TΔS, and ΔG is negative or close to zero, indicating strong binding to the molecular target.

The ΔG + ΔW value allows us to take a broader view of the properties characterizing the peptide in the complex as well as the unbound one. This value for light1 and 2 peptides further confirms their weak affinity to HVEM. In the case of light4, summing these values yields a negative value. Although this peptide does not bind HVEM strongly according to ΔG, forming the complex significantly reduces the internal work performed by the peptide. This decrease in internal work largely compensates for the low affinity, as confirmed by the SMD results. In the case of light3, we can observe a positive ΔW and the resulting positive ΔG + ΔW. A decrease in this property could potentially have a positive effect on the peptide’s affinity for HVEM, but as we observe in the case of its analogue light3_E178L, the attenuation of the negative effect of ΔW as a result of glutamic acid substitution is also compensated by an increase in ΔG. Similar conclusions can be drawn on the basis of the values of Fmax and Wtotal, where the peptides light3 and light3_E178L are significantly higher than those for the other three. This property indicates that a high force is required to detach these two peptides from the molecular target, which indicates their high affinity for HVEM and thus potentially high inhibitory properties.

On the basis of the comparison of both short and long simulations, we can see that in the case of long simulations, the standard deviations between the three dynamics are many times higher than in short simulations, which indicates the loss of stability of the complex in the course of the simulation progress. In addition, the main conclusion from the comparison of thermodynamic properties determined on the basis of short and long simulations is the fact that, in the course of simulation, the change in internal work increases significantly in the case of light1 and 4 peptides. This indicates a decreasing difference in the internal work performed by the peptide bound in the complex and the free peptide, which may be related to the increasing lability of compounds bound during the simulation. Comparing the values obtained during these studies to the values obtained in our previous studies, we can observe a similar trend, according to which peptides in which the enthalpic contribution dominating over the entropic contribution, and with the highest Wtotal and Fmax values showed the highest theoretical affinity for the molecular target determined by experimental methods. This correlation confirms the high efficiency of the analyses and their correlation with experimental results. Short molecular dynamics simulations, followed by MMGBSA calculations, revealed that light5 exhibited energy very close to zero, which indicate that during simulations it dissociate from the protein, which exclude it from further analysis. Among the remaining four peptides, light3 exhibited the most promising binding characteristics, warranting further investigation. MMGBSA decomposition indicated that E176 and E178 might have negatively impact for the stability of the HVEM/light3 complex. In order to improve the stability of peptide/protein complex they were substituted by lysine, leucine and glutamine in various configurations, which enabled us to design 9 analogues of the light3. Light3_E178L demonstrated an enhanced affinity for its molecular target. A comprehensive set of theoretical analyses performed for peptides indicated that both light3 and its analogue, light3_E178L, had the highest theoretical affinity for HVEM. The significant amount of work and force required to separate these peptides from HVEM suggested strong interactions between peptides and protein. In contrast, light1 and light2 did not exhibit significant force or work leap during SMD, implying weaker affinity toward HVEM. It should also be noted that the peptide light3, which is most strongly bound to HVEM, and light5, which does not form a stable complex with HVEM, differ only in the position of the disulfide bond. The amino acids substituted by cysteines in both tested peptides are not crucial for the interaction with HVEM, what strongly confirmed our previous observation, that the position of disulfide bond is crucial for the interaction of molecular target. The Wtotal and TΔS strongly suggest that the sequence could be further optimised by introduction of additional rigidity, for example by change of disulfide bond position.

### The affinity of the selected peptides to HVEM determined by SpS

**Fig. 7:**
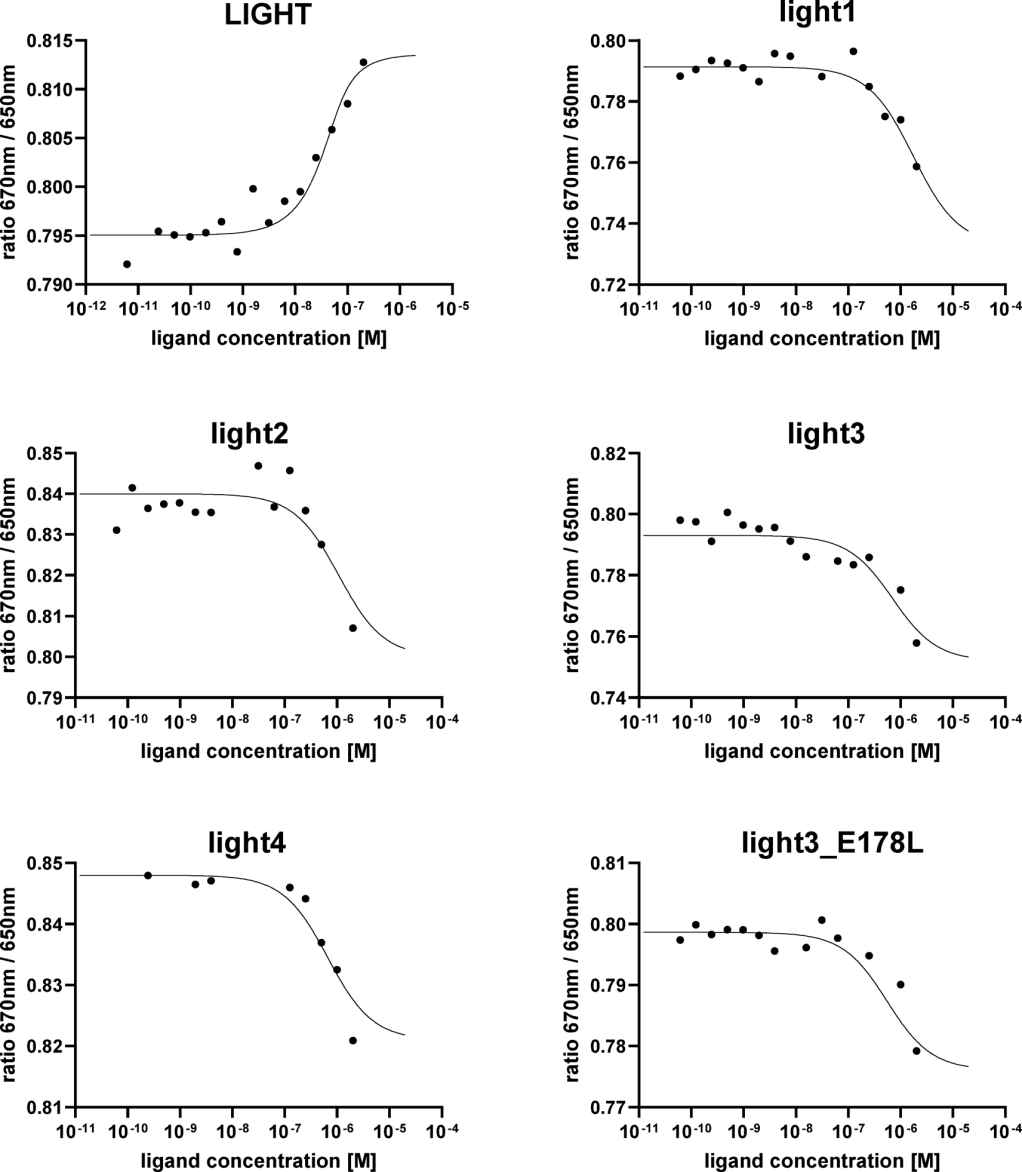
The spectral shift dose-response curves for the interactions between HVEM and LIGHT protein and synthesized peptides.

Based on the computer analyses conducted for the designed peptides in complex with the HVEM, five compounds – peptides light1, light2, light3, light4, and light3_E178L were selected for experimental validation and their affinity to HVEM was evaluated using SpS.Fluorescence intensity was measured at 670 and 650 nm (Figure S4). The apparent dissociation constants (Kd) determined for the peptide/HVEM complexes ranged from 1.72 µM for light4 to 0.58 µM for light3 (Table 2). Corresponding dose-response spectral shifts are presented in Fig. 7. Due to the fact that peptides at higher concentrations aggregated, it was not possible to fully determine the Kd. Therefore, only the apparent Kd values were determined and were used for HVEM binding strength comparison. Note that apparent Kd is only approximate value and should be used only for qualitative comparison. Among the tested peptides light3 and its analogue light3_E178L exhibited the highest binding affinities toward HVEM, even stronger than the LIGHT protein. These results strongly correlate with the data obtained from computational analyses.

**Table 2:** Affinity of the selected LIGHT-derived peptides to HVEM designated by SpS. Results are shown for experiments performed in triplicate. Data are presented as mean ± SD.

No.	Compound name	Apparent K_d_ (M)	SD
	LIGHT	0.92 × 10^−6^	0.13 × 10^−6^
1	light1	1.68 × 10^−6^	1.01 × 10^−6^
2	light2	1.38 × 10^−6^	0.39 × 10^−6^
3	light3	0.58 × 10^−6^	0.17 × 10^−6^
4	light4	1.72 × 10^−6^	0.30 × 10^−6^
5	light3_E178L	0.71 × 10^−6^	0.15 × 10^−6^

Despite the fact that the light3 and light3_E178L reveal very high binding affinity, the HVEM-derived fragments previously designed and synthesized, targeting the HVEM/LIGHT complex, exhibited even lower dissociation constants compared to the corresponding LIGHT fragments, indicating stronger binding to the molecular target (Ciura et al. submitted). It is also noteworthy that Kd values for the HVEM/LIGHT complex differ significantly depending on the proteins used in experiments and the measurement techniques applied. The Kd previously determined by us using the SpS technique indicated that LIGHT binds to the HVEM protein with a Kd of 0.92 µM. However, other research groups have also investigated this interaction, reporting Kd values ranging from 18.7 nM^11^ to 1.5 nM^41^.

### Peptides inhibitory properties assessed by competitive ELISA

**Fig. 8:**
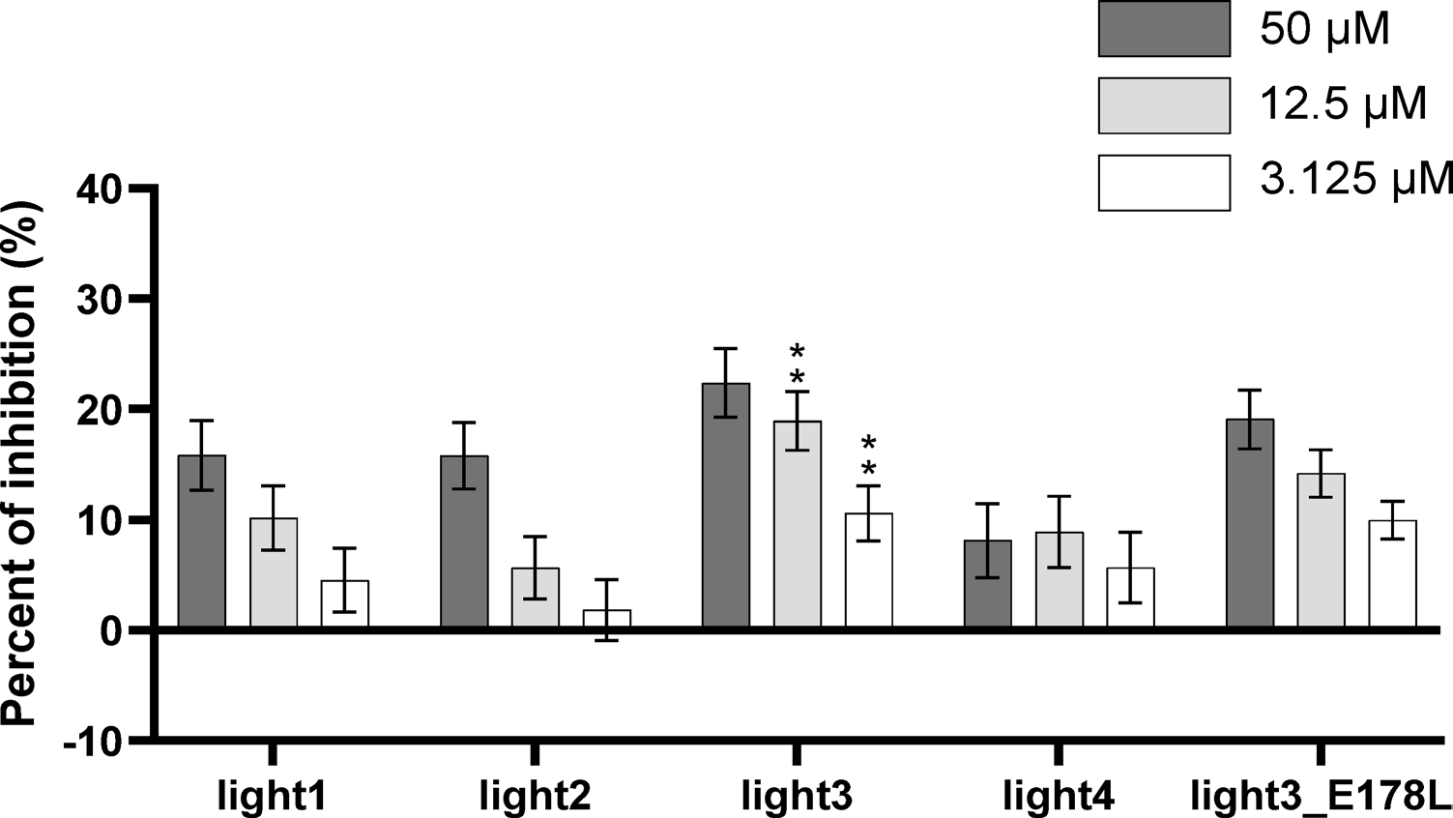
The inhibitory properties of LIGHT-derived peptides toward the formation of the HVEM/LIGHT complex determined by ELISA. The results are shown for two experiments performed independently in triplicate. Data are depicted as mean with SD (mean +/- SD). Statistical analysis was performed using one-way analysis of variance (ANOVA) followed by Dunnett’s post-hoc test. ****: *p* < 0.0001, ***: *p* < 0.001, **: *p* < 0.01, *: *p* < 0.05.

To assess the inhibitory properties of the peptides the ELISA was conducted. Based on the obtained results, it can be concluded that the inhibitory properties of the peptides are strongly correlated with their affinity to HVEM as determined by theoretical methods, as well as with those measured by SpS. The weakest inhibitory effects were observed for light4 with inhibition remaining below 10% across all tested concentrations. In contrast, the peptides light3 and its analogue light3_E178L exhibited the most significant ability to disrupt HVEM/LIGHT complex formation, with inhibition reaching 22% and 19%, respectively, at the highest concentration tested (Fig. 8).

However, the obtained peptides exhibited significantly weaker inhibitory properties compared to the HVEM-derived fragments we previously studied. The highest blocking activity was observed for CRD2e_K54E and CRD2(39–73)e, which inhibited the HVEM/LIGHT interaction by approximately 38% and 41%, respectively, at the highest concentration tested (Ciura et al. submitted). In our earlier studies, we also demonstrated that commercially available anti-HVEM antibodies inhibit HVEM/LIGHT binding by approximately 75% at a concentration of 1 µg/well (10 ng/mL)^34^. The obtained results highlight the need for further structural optimization of LIGHT-derived peptides to enhance their inhibitory properties.

### Peptides inhibitory properties measured by cellular assay

The inhibitory effects of the peptides were also evaluated using cell line-based platform, but before performing this tests, the stability of peptides in medium used in co-culture and cytotoxicity towards cell lines used in the experiments had to be determined. After 24 h of incubation (the time when the peptides are incubated with cells), there is a clear difference in stability between peptides based on one of the interaction centres (light1 and 2 – stability is 88% and 77% respectively) and between those based on the other centre (light3, 4 and 3_E178L – stability ranging from 49% to 29%). However, all peptides, despite some degradation, are present in the sample during the experiment (Figure S5). The cytotoxicity studies indicate that the analyzed peptides do not have significant effect on cell viability up to a concentration 50 µM (Figure S6) and this concentration was chosen as the highest in the cellular studies.

Reporter cells expressing the HVEM protein (JE6.1-NF-κB::eGFP HVEM) and T cell stimulators expressing LIGHT (TCS LIGHT) were utilized in cellular assays and the principle of this cell-based assay has been described in detail in previous publication^21^. In short, when the peptides inhibited the HVEM/LIGHT interaction, a reduction in NF-κB activation was observed, accompanied by a corresponding decrease in enhanced green fluorescent protein (eGFP) levels. The percentage of reporter cell activation was calculated relative to a control condition, which was reporter cell co-cultured with TCS without peptides.

**Fig. 9:**
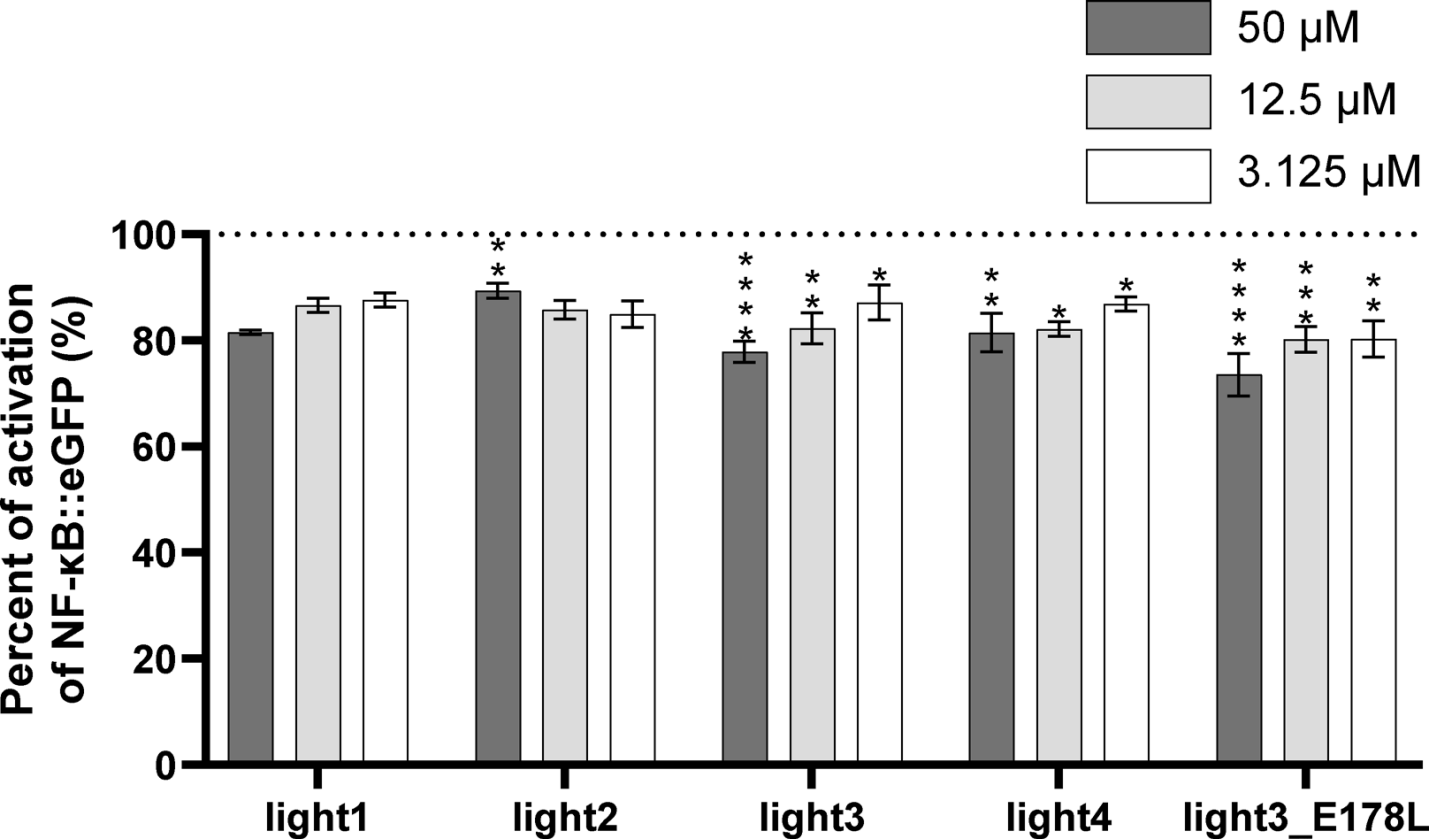
The percentage of activation of the NF-κB::eGFP signal pathway, which is corelated with HVEM/LIGHT complex formation in the presence of LIGHT-derived peptides determined by a cellular-based assay. The HVEM reporter cells were stimulated with TCS LIGHT in the absence or presence of the peptides. HVEM/LIGHT stimulation in the absence of peptides was set to 100% activation. The results are shown for three experiments performed independently in triplicate. Data are depicted as mean with SD. Statistical analysis was performed using one-way ANOVA followed by Dunnett’s post hoc test. ****: *p* < 0.0001, ***: *p* < 0.001, **: *p* < 0.01, *: *p* < 0.05.

A slight reduction in HVEM/LIGHT binding was observed in the presence of all tested peptides (Fig. 9). Light3 and light3_E178L peptides exhibited the best inhibitory effects, leading to a decrease in reporter cell activation levels to 78% and 74%, respectively, at the highest tested concentration. These findings are in agreement with previous results obtained from ELISA, which further confirmed the HVEM/LIGHT-complex-formation-disruptive properties of these peptides.

It should, however, be noted that the inhibitory properties of peptides based on the LIGHT protein are notably weaker than those of the HVEM fragments studied by us previously (Ciura et al. submitted). Two peptides, namely CRD2e_K54E and CRD2(39–73)e, have significantly higher blocking effects than the LIGHT-derived peptides, and inhibit the HVEM/LIGHT complex formation in 41% and 46%, respectively, at the highest concentration used. This may suggest that targeting the LIGHT protein is a more effective method of blocking the HVEM/LIGHT complex than targeting HVEM. All theoretical and experimental data suggest, that designing peptides when two molecules (two LIGHT monomers) are involved in complex formation is more challenging then when only single molecule (HVEM) is involved. Moreover, it should be noted that the LIGHT-derived peptides are prone to degradation, which may also affect their observed ability to inhibit protein binding in cell-based assays. To address this issue, structural modifications are necessary to enhance their resistance to degradation, such as introducing non-natural amino acids into the sequence or substituting L-amino acids with their D-enantiomers. The LIGHT-derived peptides are linear, whereas our previously studied and described immune checkpoint inhibitors^38^ were primarily stabilized by disulfide bonds, which, as confirmed by literature data^42^, improve peptide stability. Therefore, peptide stability represents a significant limitation of the present study.

## Conclusion

Our study contributes to the growing field of immune checkpoint modulation and peptide-based therapeutics by identifying and characterizing LIGHT-derived peptides as potential inhibitors of the HVEM/LIGHT complex. During our research the peptides light3 and its analog light3_E178L exhibited favorable binding characteristics and promising inhibitory properties. All our in silico and experimental studies indicate promising binding properties of light3 and light3_E178L. Theoretical studies suggest that light3 sequence could be further improved by altering position of disulfide bond, which is our plan for the next studies. All these results provide a solid foundation for further structural optimization and preclinical evaluation, supporting their future development as novel immune-modulating agents.

Nevertheless, several limitations of the present study should be acknowledged. In particular, the employed in vitro assays, such as SpS, ELISA, and cell-based tests, do not fully capture the complexity of in vivo biological systems. Additionally, peptides often exhibit limited stability and bioavailability, which may affect their therapeutic potential. Comprehensive evaluation of the immunomodulatory potential of the identified peptides requires further investigation. This includes in vitro assays to assess their effects on T cell activation, proliferation, differentiation, and cytokine production, as well as in vivo experiments employing advanced models, such as mice with induced autoimmune diseases. Such approaches are essential to fully elucidate the therapeutic efficacy and clinical relevance of these peptides.

## Materials and methods

The overall workflow of this study is depicted in Figure S11. The analysis commenced with the structural characterization of the HVEM/LIGHT complex. Subsequently, the designed peptides were subjected to MD simulations, and only conformationally stable complexes were retained for in-depth theoretical investigations. Computational peptide optimization was performed prior to advancing to the experimental validation stage.

### Molecular dynamics and MMGBSA analyses and thermodynamic values assessment

Analyses were conducted based on the crystal structure of the HVEM/LIGHT protein complex (PDB code 4RSU). The missing residues were added using modeller9.16^43^. The files necessary for the simulation were prepared on the basis of the tleap program using the ff19SB force field with a four-site OPC water model^44–46^. The simulations were run using pmemd.cuda from the AMBER24^47^package. The simulated systems were enclosed within a 17.5 Å layer of water, shaped as a truncated octahedron, and their total charge was neutralized by adding either Na⁺ or Cl⁻ ions. Each system underwent two stages of energy minimization: the first minimization (steepest descent) consisted of 1000 steps with restraints applied to all non-solvent atoms, while the second minimization (conjugated gradient) involved 2000 steps with restraints on all heavy atoms. This was followed by a three-phase equilibration process. Initially, the system was gradually heated from 10 K to 300 K over 10^4^ MD steps. The second phase, consisting of 10^5^ steps, was used to establish the target density. Finally, a third equilibration consisting of 4 × 10^5^ steps npt with Langevin thermostat and weak restraints on Cα and Cβ atoms was performed to ensure system stability. Based on these equilibrated setups, three independent molecular dynamics simulations were conducted, each lasting 200 ns, corresponding to 10⁸ steps with a 2 fs time step. For the selected systems longer molecular dynamics were also carried out. In this case equilibration and minimisation steps were conducted the same way, but the overall simulations were longer – 5 × 10^8^ steps of simulations (2 fs each) resulting in 1 µs of simulation. In this case we have analysed last 500 ns of these simulations. For analysis, we focused on the final 20 ns of each trajectory to minimize any influence from initial simulation convergence. The binding free energy was estimated using the MMGBSA approach, specifically employing the GB-Neck2 model with its associated atomic radii parameters^48–51^. The entropic input was also used to calculate Gibbs free energy with normal mode analysis methods for 10 frames drawn uniformly from the last 20 ns of 200 ns simulations. For longer analyses, 20 frames from the last 200 ns of the simulation were analyzed. Change of internal work was calculated by subtracting the energy calculated from the MMGBSA of the peptide not bound in the complex from the energy of the peptide in the complex.

### Steered molecular dynamics

For each of the selected peptides, simulations consisting of 20 trajectories of 80 ns each were carried out. The output files were prepared on the basis of a structure representative of each complex based on clustering. The systems were surrounded by a 70 Å layer of water. This was followed by the standard course of minimization and equilibration described in the previous paragraph. The mass center of the Cα carbons of the peptide was pulled away from the mass center of the Cα carbons of the HVEM protein. Extraction with a force constant of 10 kcal/mol/Å^2^ and pulling speed of 0.05 m/s was continued until a distance of 40 Å between the mass centres was reached. Structures near force peak were extracted from simulation and DBSCAN clustering algorithm^52^ was performed with use of cpptraj^44^.

### Peptides synthesis and purification

The peptides were synthesized using solid-phase peptide synthesis (SPPS) on a Liberty Blue synthesizer (CEM Corporation, Matthews, USA) following the Fmoc/tBu strategy. Rink Amide Pro Tide (LL) resin (CEM Corporation, Matthews, USA) with a loading capacity of 0.18 mmol/g was used. Methionine residues in synthesized peptides were replaced with norleucine to prevent their oxidation during experiments, as in our previous studies. The synthesis protocol was based on a previously described method^38^. Purification was conducted using reverse-phase high-performance liquid chromatography (RP-HPLC) on a Luna C8(2) column (250 × 4.6 mm, 5 μm, 100Å, Phenomenex, CA, USA). Prior to loading, the peptide solution was treated with a 10-fold excess of dithiothreitol (DTT) relative to the free thiol groups of cysteine residues and sonicated at 60 °C. Two solvents were used for purification: solvent A (deionized water with 0.1% TFA) and solvent B (80% acetonitrile in water with 0.1% TFA). A linear gradient from 5% to 50% of solvent B in A over 120 min was applied, with UV detection at 222 nm and 254 nm. Peptide molecular weight and purity were verified using liquid chromatography coupled with electrospray ionization, ion trap, and time-of-flight mass spectrometry (LC ESI-IT-TOF MS, Shimadzu, Kyoto, Japan). Additional purity confirmation was performed using reverse-phase ultra-high-performance liquid chromatography (RP-UHPLC) with a photodiode array detector (PDA) and an evaporative light scattering detector (ELSD-LT) (Shimadzu, Kyoto, Japan) on a Kinetex C8 column (100 × 2.1 mm, 2.6 μm, 100Å, Phenomenex, CA, USA). A gradient from 5% to 100% solvent B in A over 15 min was used.

### Formation of disulfide bond

To create the disulfide bond, peptides were dissolved in a water-methanol mixture (1:9, v/v) at concentration 40 mg/L. The pH was adjusted to 8–9 using 25% ammonia, and the solution was stirred for 7 days while simultaneously introducing compressed air to promote oxidation. Reaction progress was monitored by RP-UHPLC. Next, the methanol was evaporated, and the remaining solution was freeze-dried. The peptides were purified again using the same protocol as previously described.

### Spectral shift

SpS experiments were conducted using the Monolith X instrument (Nanotemper, Munich, Germany). Protein labelling was performed using the His-Tag Labeling Kit RED-tris-NTA 2nd Generation (Nanotemper, Munich, Germany). To prepare the fluorescent dye solution at concentration 0.1 µM, 2 µL of a 5 µM dye stock was diluted in 98 µL of deionized water. Next 90 µL of this solution was mixed with 90 µL of 100 nM HVEM-His protein (ACROBiosystems, Newark, USA) dissolved in PBS-T (phosphate buffer with 0.05% Tween-20 and 0.3 M NaCl, pH 7.4) and incubated for 30 min at room temperature. Next, 10 µL of the labelled protein was mixed with 10 µL of peptide solutions at concentrations ranging from 4 µM to 12.2 pM. This resulted in a final protein concentration of 50 nM and peptide concentrations ranging from 2 µM to 6.1 pM. The mixtures were loaded into Monolith X-compatible capillary tubes, and fluorescence ratios (670/650 nm) were measured at 40% excitation power and average IR laser power at a temperature of 25 °C. Data were analyzed using MO.Control 2 software (version 2.5.4, NanoTemper, Munich, Germany). Each experiment was performed in triplicate.

### Enzyme-linked immunosorbent assay

ELISA was conducted using transparent polystyrene 96-well plates (Brand, Wertheim, Germany). Wells were coated with HVEM-His protein at concentration 6 µg/mL dissolved in PBS-T (100 µL per well) and incubated overnight at 4 °C. Unbound sites on the wells by HVEM were blocked with 5% bovine serum albumin (BSA) in PBS-T (200 µL per well). Peptides at three concentrations 50 µM, 12.5 µM, and 3.125 µM dissolved in PBS-T were added and incubated (200 µL per well). These peptide concentrations were selected because they did not exhibit cytotoxicity toward the cells used in subsequent experiments. Subsequently, LIGHT-Fc (ACROBiosystems, Newark, USA) at concentration 2 µg/mL (100 µL per well) was introduced and incubated. In the next step horseradish peroxidase (HRP)-conjugated goat antibody (Bio-Rad, Hercules, USA) at dilution 1:3000 in PBS-T (100µL per well) was applied. After each step, wells were washed five times with 200 µL of PBS-T. Incubations were carried out at 37 °C for 2 h, except for the antibody, which was incubated for 1 h. Finally, 200 µL of TMB substrate (Thermo Fisher Scientific,Waltham, MA, USA) was added, and after 15 min of incubation in the dark, absorbance was measured at 492 nm and 650 nm using an Infinite M200 Pro reader (Tecan, Switzerland). The negative control consisted of PBS-T buffer without peptides, while the positive control was mouse anti-human HVEM antibodies (1 µg/well, Bio-Rad, Hercules, CA, USA; #MCA6072GA). Each experiment was performed in three biological replicates, each consisting of three technical replicates and data were analyzed using one-way ANOVA with Dunnett’s post-hoc test in GraphPad Prism (San Diego, USA).

### Cellular assays

The experiments utilized the JE6.1-NF-κB::eGFP HVEM (modified Jurkat), TCS LIGHT, TCS Crtl and TCS CD86 Ctrl cell lines (modified BW5147) which have been described in earlier studies^57,58^. Jurkat E6.1 cell line (describe in Supplementary Materials) was purchased from Cell Line Service GmbH, while the JE6.1-NF-κB::eGFP HVEM, TCS LIGHT, TCS Crtl and TCS CD86 Ctrl cell lines are property of the laboratory of Prof. Peter Steinberger, Institute of Immunology, the Medical University of Vienna, Vienna, Austria. Cell lines were cultured in RPMI 1640 medium (Sigma Life Sciences, USA) supplemented with 1% penicillin and streptomycin with the addition of 10% heat-inactivated fetal bovine serum (FBS, Sigma Life Sciences, USA). For the cellular assays, 25 µL of peptide solutions at three concentrations (200, 50, and 12.5 µM) were incubated with 50 µL of JE6.1-NF-κB::eGFP HVEM cell suspension (50 × 10⁴ cells per tube) for 1 h at 37 °C. The tubes were then centrifuged (500 × g, 3 min), the supernatant containing unbound peptides was removed, and 75 µL of fresh medium was added. The peptide-bound reporter cells were transferred to a 96-well plate (Sarstedt, Germany) and 25 µL of TCS LIGHT cell suspension (2 × 10⁴ cells per well) was added to each well. The plates were incubated for 24 h at 37 °C and 5% CO₂. After incubation, the plates were centrifuged (800 × g, 3 min), and 6 µL of APC-mCD45 antibody was added to each well to exclude TCS cells from the analysis. The plates were incubated for 20 min at 4 °C, washed with FACS buffer (PBS, 5–10% FBS, 0.1% NaN₃), and resuspended in 50 µL of fresh FACS buffer. eGFP expression was measured using a CytoFLEX flow cytometer (Beckman Coulter, USA). Data were analyzed using FlowJo v10.10 (FlowJo LLC, USA) and GraphPad Prism (San Diego, USA) with univariate ANOVA and Dunnett’s post-hoc test. Control experiments were performed using cells not expressing the LIGHT protein (TCS CD86 Ctrl and TCS Ctrl) to ensure the specificity of the peptide effects. The results were represented as an average. Each analysis was performed in three biological replicates, each consisting of three technical replicates.

### Figure preparation

Figures 1 and 5 were prepared with Pymol version 2.6, Figs. 2, 3, 4, 6, 7, 8 and 9 were prepared with GraphPad Prism version 10.4.

## Supplementary Information

Below is the link to the electronic supplementary material.

Supplementary Material 1

## Data Availability

The datasets generated and/or analysed during the current study are available in the University of Gdańsk: (https:/repozytorium.bg.ug.edu.pl/info/researchdata/UOG8e9011f720ef48bbbd342089280d3168) DOI:10.71853/5dj5-sp15.
