# Supplementary material for: Evaluation of LIGHT-derived peptides to disrupt the HVEM/LIGHT immune checkpoint
Source: Sci Rep. 2025 Nov 17;15:40095. doi: 10.1038/s41598-025-23946-4 (PMC12623744; doi:10.1038/s41598-025-23946-4)
Supplement: Supplementary file 1 — Supplementary Material 1 [file 41598_2025_23946_MOESM1_ESM.pdf]

Supporting Information for:
Evaluation of LIGHT-Derived Peptides to Disrupt the HVEM/LIGHT Immune Checkpoint.

Piotr Ciura[1], Simon Gumpelmair[2], Sylwia Rodziewicz-Motowidło[3], Peter Steinberger[2], Marta Spodzieja[3], Adam K. Sieradzan*[1]

[1] *Faculty of Chemistry, Department of Theoretical Chemistry*
University of Gdańsk,
Wita Stwosza 63, 80-308 Gdańsk, Poland

[2] *Division of Immune Receptors and T cell Activation, Institute of Immunology, Centre for Pathophysiology, Infectiology and Immunology,*
Medical University of Vienna
Lazarettgasse 19, 1090 Vienna, Austria

[3] *Faculty of Chemistry, Department of Biomedical Chemistry*
University of Gdańsk
Wita Stwosza 63, 80-308 Gdańsk, Poland

* *Corresponding authors: adam.sieradzan@ug.edu.pl*

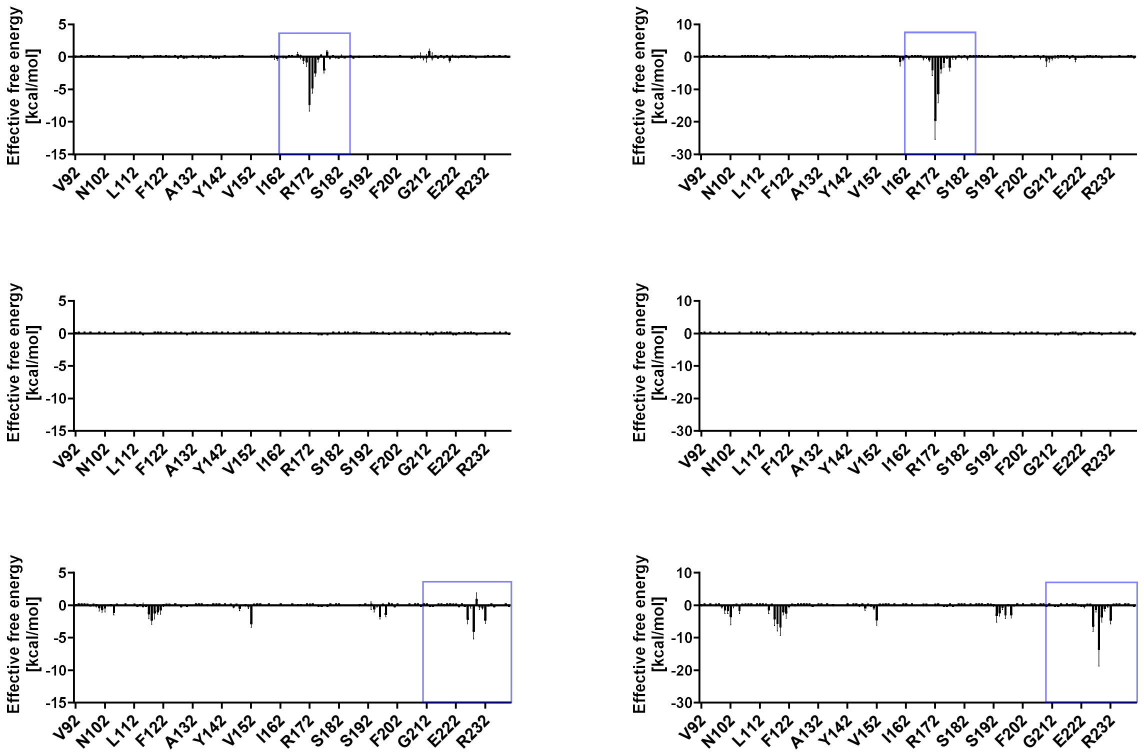

Figure S1. *MM-GBSA per residue (left) and pairwise (right) effective binding energy decomposition results for three LIGHT protein monomers. The fragments on the basis of which the peptides were designed are marked with a blue rectangle. Results are shown for 3 independent trajectories as mean ± SD.*

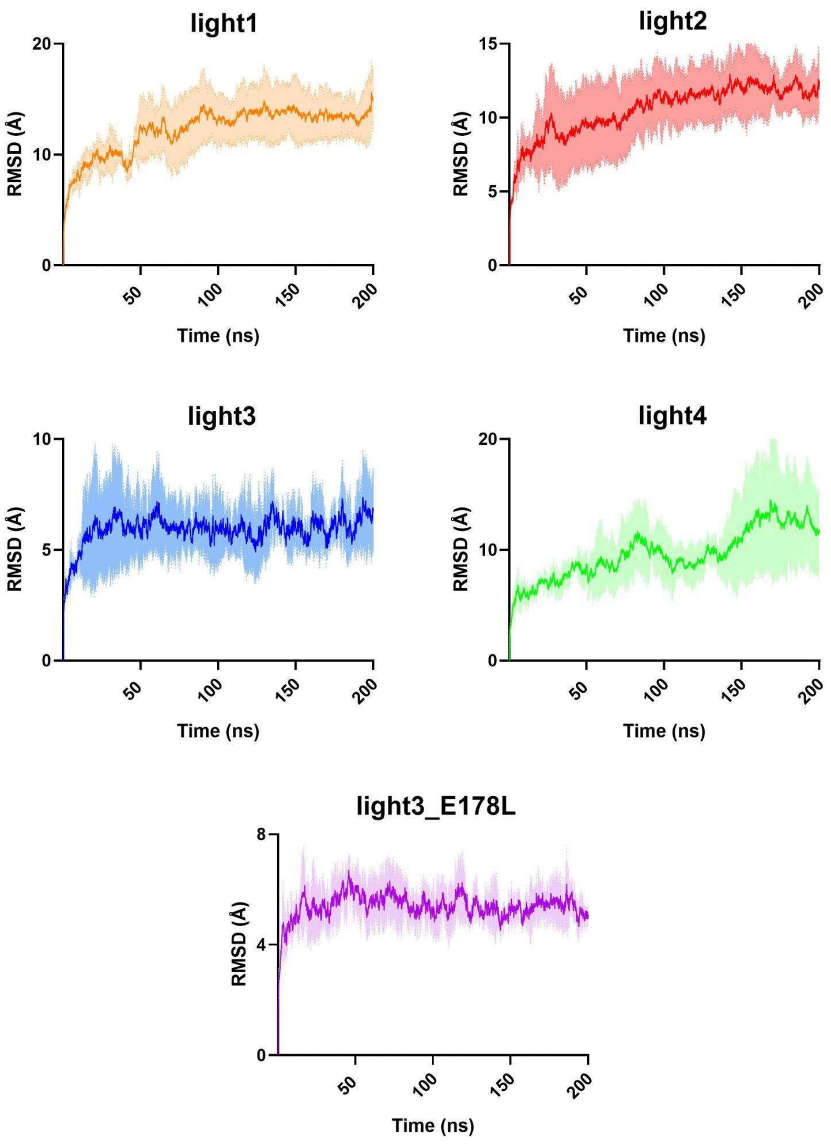

Figure S2. *Root-Mean-Square Deviation plot of light1-4 peptides and the light3_E178L analogue based on three 200 ns trajectories. Results are presented as mean (bold line) ± standard deviation (shaded area). The flattening of the curves in the last stage of the simulation indicates that the system has reached equilibrium.*

Table S1. *Top 10 amino acid residues of the LIGHT protein with the highest summarised energy contribution for the formation of the HVEM/LIGHT complex, determined on the basis of pairwise decomposition of MM-GBSA analysis together with HVEM protein residues constituting the main interaction partner of a given residue. The letters A and B after residue numer distinguish between the individual monomers of the LIGHT protein. Results are shown for 3 independent trajectories as mean ± SD.*

No.	LIGHT residue	HVEM residue	Theoretical interaction energy [kcal/mol]
1	R 172 A	P 48	-19.81±5.53
2	R 228 B	D 100	-13.71±4.97
3	Y 173 A	H 86	-11.52±2.61
4	G 119 B	K 92	-6.75±2.46
5	R 226 B	D 100	-6.62±1.42
6	L 118 B	Q 95	-5.68±2.18
7	R 232 B	D 131	-4.67±1.06
8	V 152 B	M 103	-4.63±1.53
9	Q 117 B	K 92	-4.34±1.89
10	P 171 A	P 48	-4.04±1.76

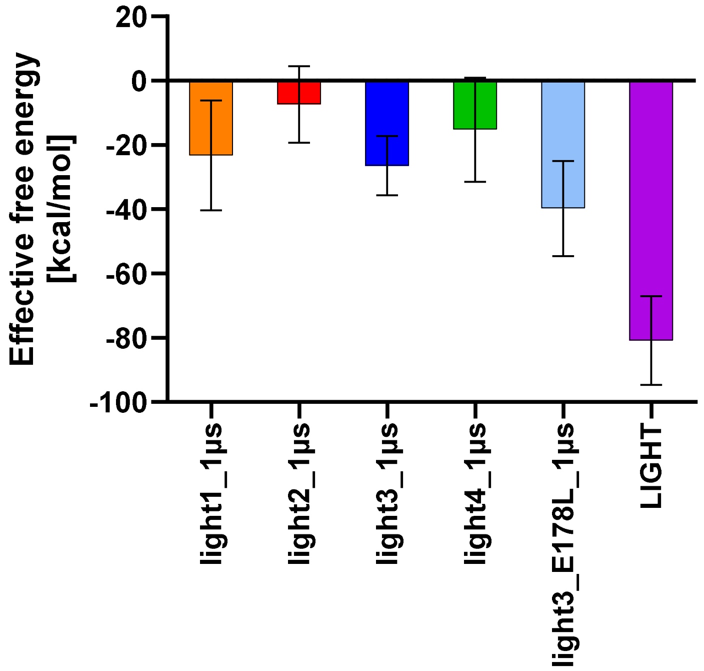

Figure S3. *Diagram of effective binding free energy for 1000 ns calculations based on MM-GBSA of initial peptide structures and the light3 peptide analogue. The results are presented as mean ± SD based on analyses from three independent trajectories.*

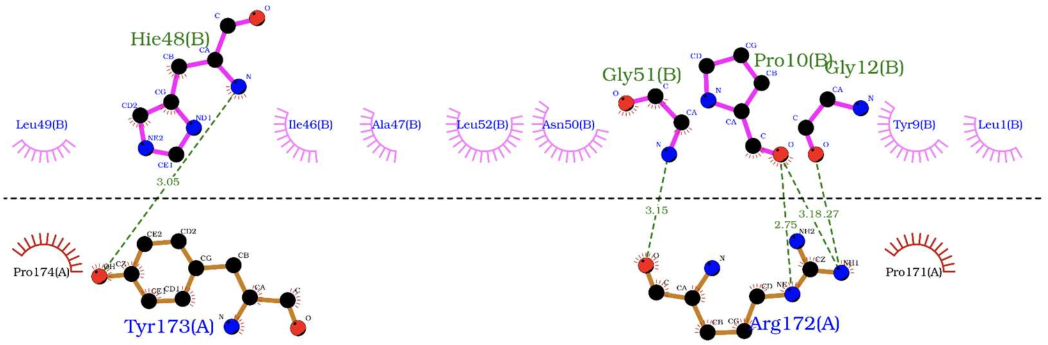

light3

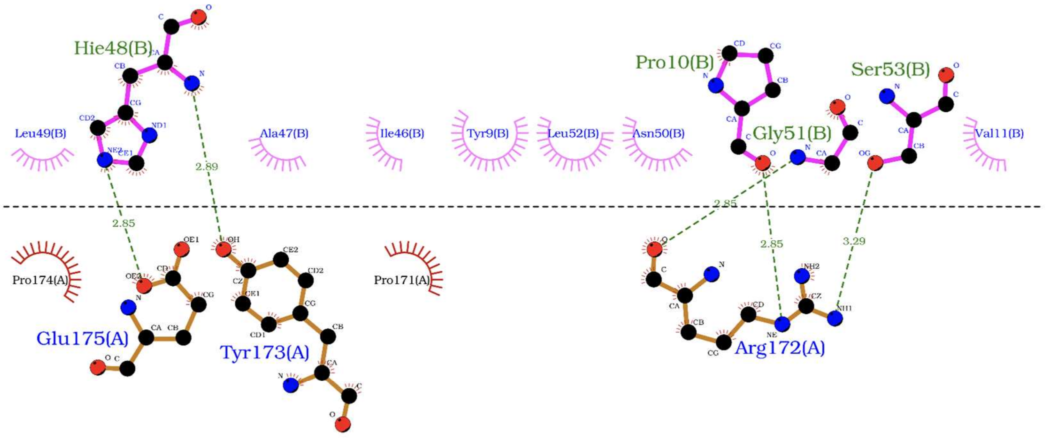

light3_E178L

Figure S4. *Analysis of interaction performed with LigPlus for light (top) and light3_E178L.*

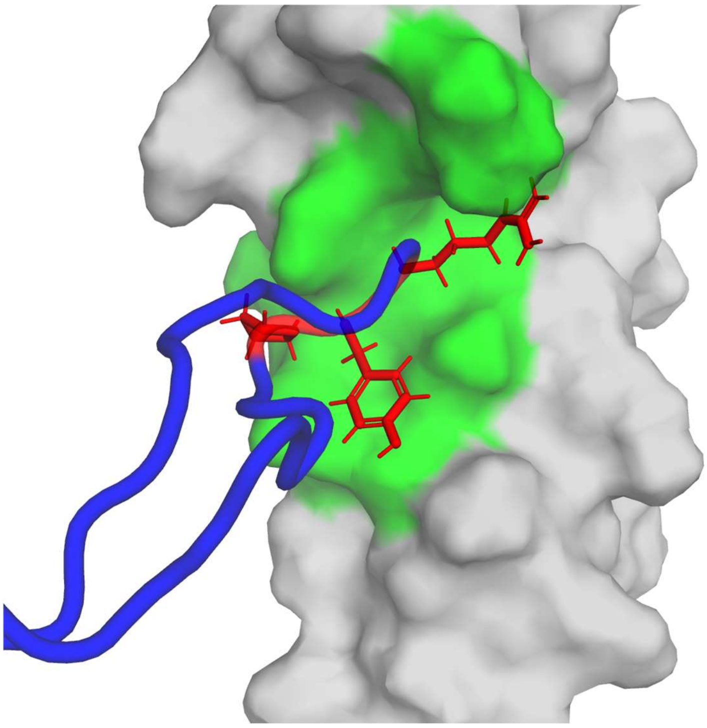

Figure S5. *Representative structure of the light3 peptide (blue cartoon) and the HVEM protein (gray area) compex. Residues R172, Y173, and P174 (red) which were determined during the analysis to have a significant energy contribution to the formation of the peptide/protein complex interact with the groove formed by L1, Y9-V11, and I71-L77 of the HVEM protein (green area).*

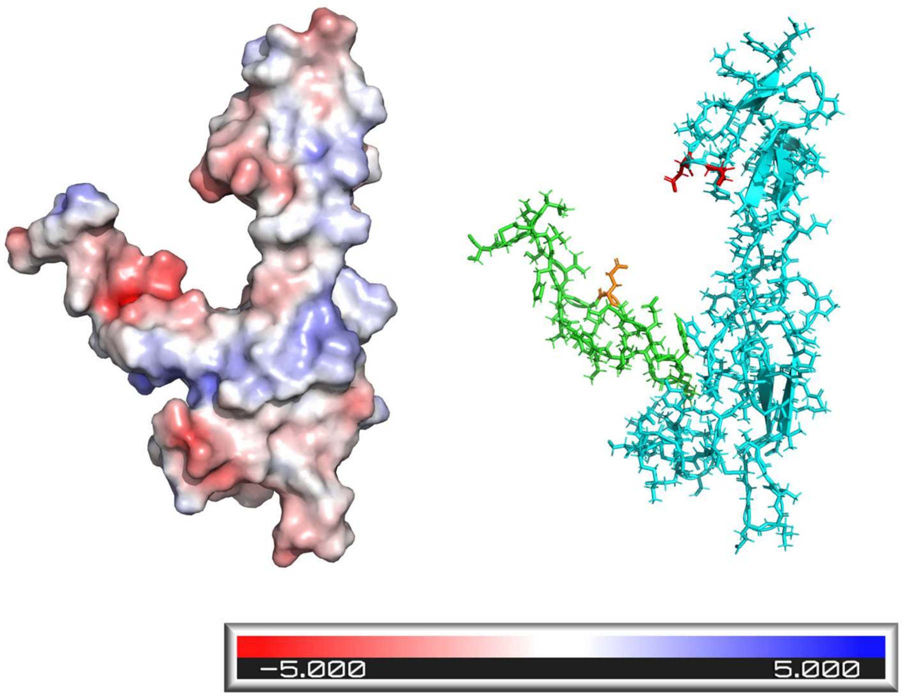

Figure S6. *Structural representation of the light3–HVEM complex. The left panel shows the electrostatic surface potential of the complex, calculated using APBS, with the potential isosurface color scale displayed at the bottom. The right panel depicts a cartoon and stick representation, with light3 in green and HVEM in cyan. The side chains of light3 Glu17 (orange), and HVEM Asp97, and Asp99 (red) are highlighted.*

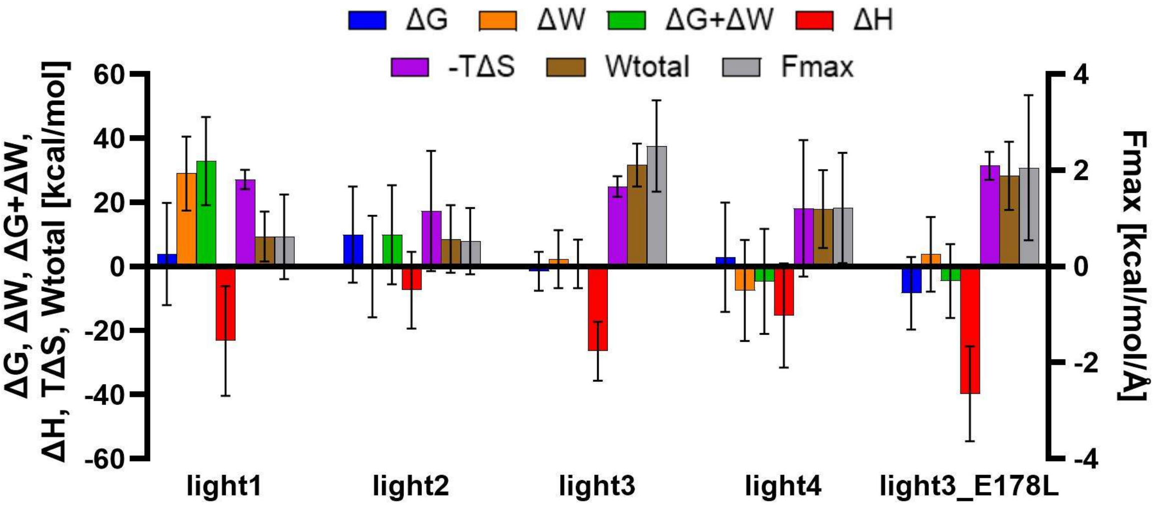

Figure S7. *Diagram presenting change of: Gibbs free enthalpy, internal work, sum of Gibbs free enthalpy and internal work, free enthalpy, entropy, total work and maximum force (right Y axis) for the four starting peptides and the light3 peptide analogue for long (1µs) simulations.*

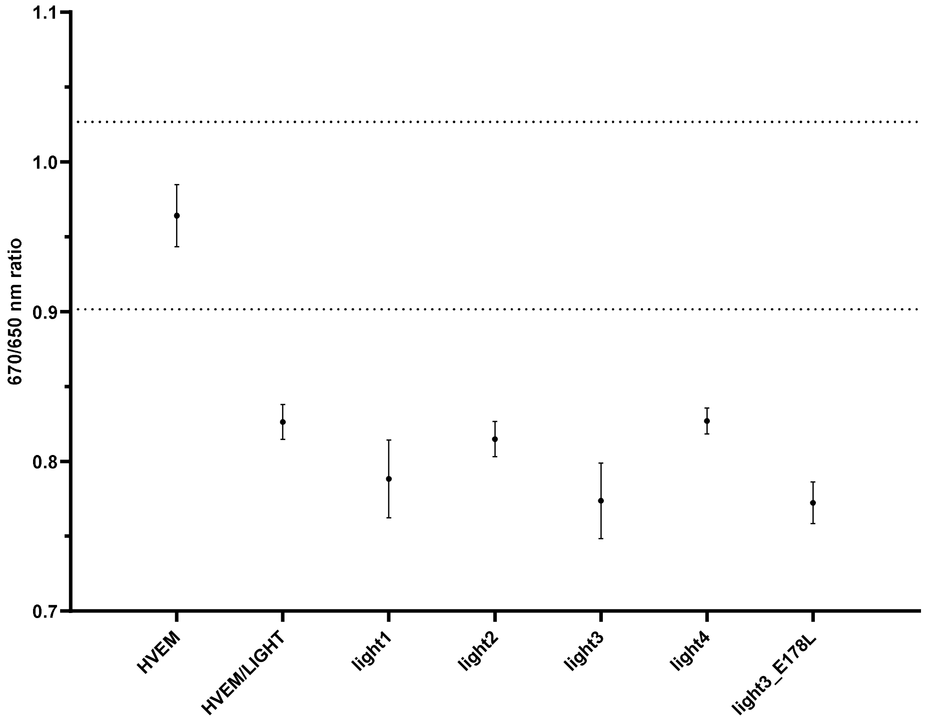

Figure S8. *SpS binding check experiments for selected LIGHT-derived peptides and HVEM compared to negative control (HVEM protein without any ligand). Dotted line represents three times the standard deviation range. The graph shows results from 3 independent experiments. Results are presented as mean with SD.*

Peptides stability in medium

Peptide stability was assessed using the supernatant from the Jurkat E6.1 cell line. The supernatant was collected after 24 hours of cell incubation (1 million cells in 10 mL of medium) under standard culture conditions. The cells were centrifuged (3000×g, 3 minutes), and the supernatant was transferred to a clean container. A 50 µL aliquot of peptide solution in water was added to 200 µL of supernatant, achieving a final peptide concentration of 1.5 mg/mL. The mixture was incubated at 37°C with continuous stirring. Samples were taken at 0 and 24 hours, frozen, and stored until analysis. Prior to analysis, the samples were thawed on ice, mixed with a 4-fold excess of ethanol (v/v), and centrifuged (24,270×g, 20 minutes, 4°C). The supernatant was evaporated and reconstituted in 120 µL of a solution containing 13% acetonitrile in water with 0.1% TFA (v/v). The samples were analyzed by reverse-phase high-performance liquid chromatography (RP-HPLC) on a Kromasil C8 column (250 nm × 4.6 mm, 5 µm) using a gradient of 5% to 100% solvent B over 60 minutes. Peptide amounts were quantified by

comparing the peak areas of the peptides in the appropriate samples to those of peptide dissolved in water at time 0. All experiments were conducted in triplicate.

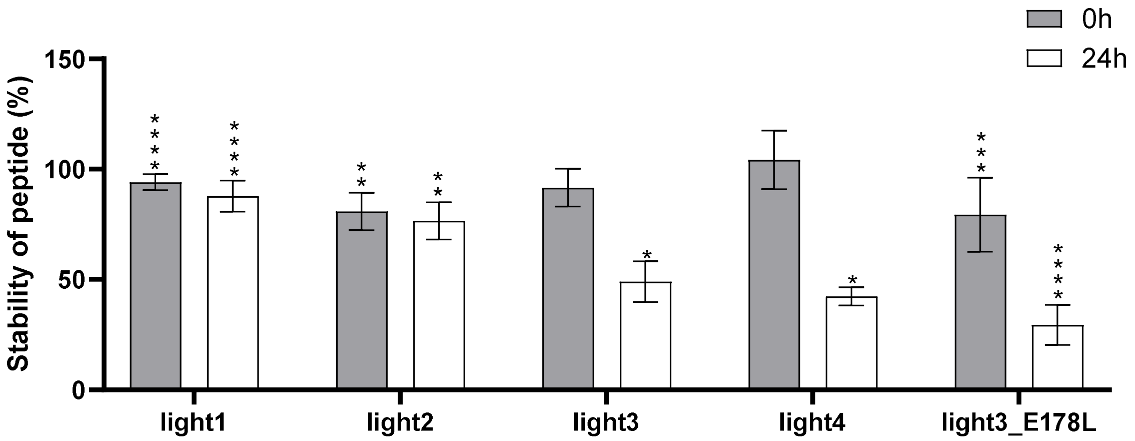

Figure S9. *Stability of peptides in Jurkat E6.1 cell supernatant at 0 and 24 h of incubation compared to the control (peptide dissolved in H_2O at time 0). The results are shown for three independent experiments. Data are depicted as mean with SD (mean +/- SD). Statistical analysis was performed using one-way ANOVA followed by the Dunnet post hoc test. ****: $p < 0.0001$, ***: $p < 0.001$, **: $p < 0.01$, *: $p < 0.05$.*

Peptides cytotoxicity

The cytotoxic effects of the peptides were evaluated on two cell lines: Jurkat E6.1 and TCS-Ctrl (a modified BW5147 cell line). The Jurkat E6.1 cells were obtained from Cell Line Service GmbH, while the TCS-Ctrl line was provided by Prof. Peter Steinberger's laboratory at the Medical University of Vienna. Both cell lines were maintained in RPMI1640 medium (Sigma-Aldrich, USA) supplemented with 1% antibiotics (penicillin and streptomycin) and 10% fetal bovine serum (FBS), and cultured at 37°C in a 5% CO_2 atmosphere. For the cytotoxicity assay, white 96-well plates (Thermo Fisher Scientific, USA) were seeded with 40 µL of cell suspension (1×10^4 TCS cells/well or 2×10^4 Jurkat E6.1 cells/well) in culture medium. Peptide solutions were prepared in the medium at concentrations ranging from 100 µM to 6.25 µM, and 40 µL of each solution was added to the wells, resulting in final peptide concentrations of 50 µM to 3.125 µM in a total volume of 80 µL per well. The cells were incubated with the peptides for 24 hours at 37°C and 5% CO_2. After incubation, the plates were allowed to cool to room temperature for 10 minutes, followed by the addition of 80 µL of CellTiter-Glo reagent

(Promega, USA) to each well. Luminescence was measured after 15 minutes using a CLARIOstar plus reader (BMG Labtech, Germany). Cell viability was calculated as a percentage relative to peptide-free control cells which consisted of cells incubated under the same conditions but without the addition of peptides. Cell viability in this control group was defined as 100%, and the effects of the peptides on cell viability were assessed relative to this baseline.The results represent the average of three independent experiments, each performed in triplicate. Data were analyzed using GraphPad Prism 9 (San Diego, USA) with univariate ANOVA and Dunnett’s post-hoc test.

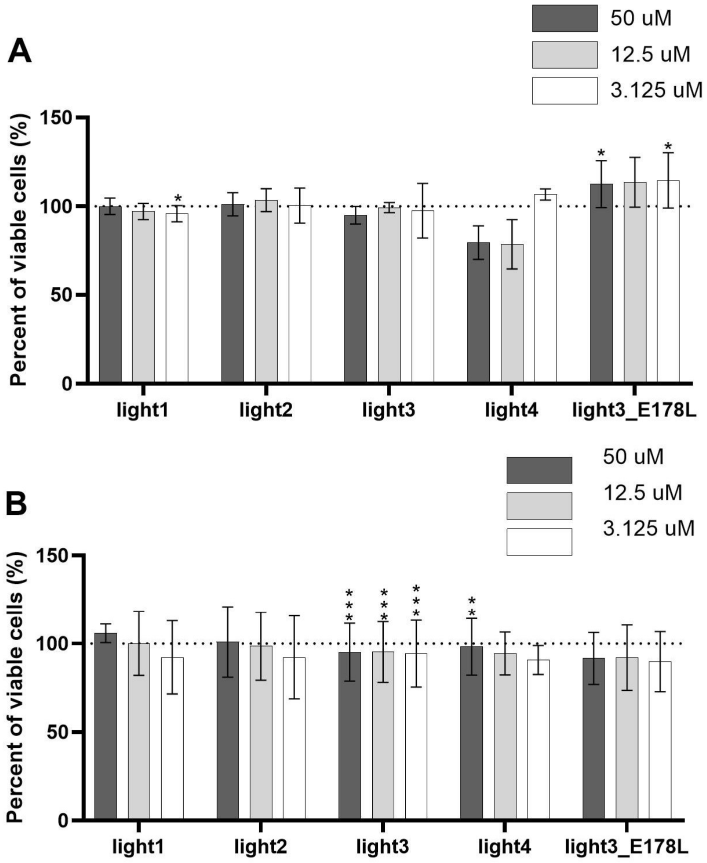

Figure S10. *Cell viability assay used to define the influence of LIGHT-derived peptides on A) Jurkat E6.1, B) TCS Ctrl cell lines after incubation for 24 hours. The results are shown for three experiments performed independently in triplicate. The dashed line indicates the value for the control (incubation of cells without the presence of peptide)Data are depicted as mean with SD (mean +/- SD). Statistical analysis was performed using one-way ANOVA followed by the Dunnet post hoc test. ****: $p < 0.0001$, ***: $p < 0.001$, **: $p < 0.01$, *: $p < 0.05$.*

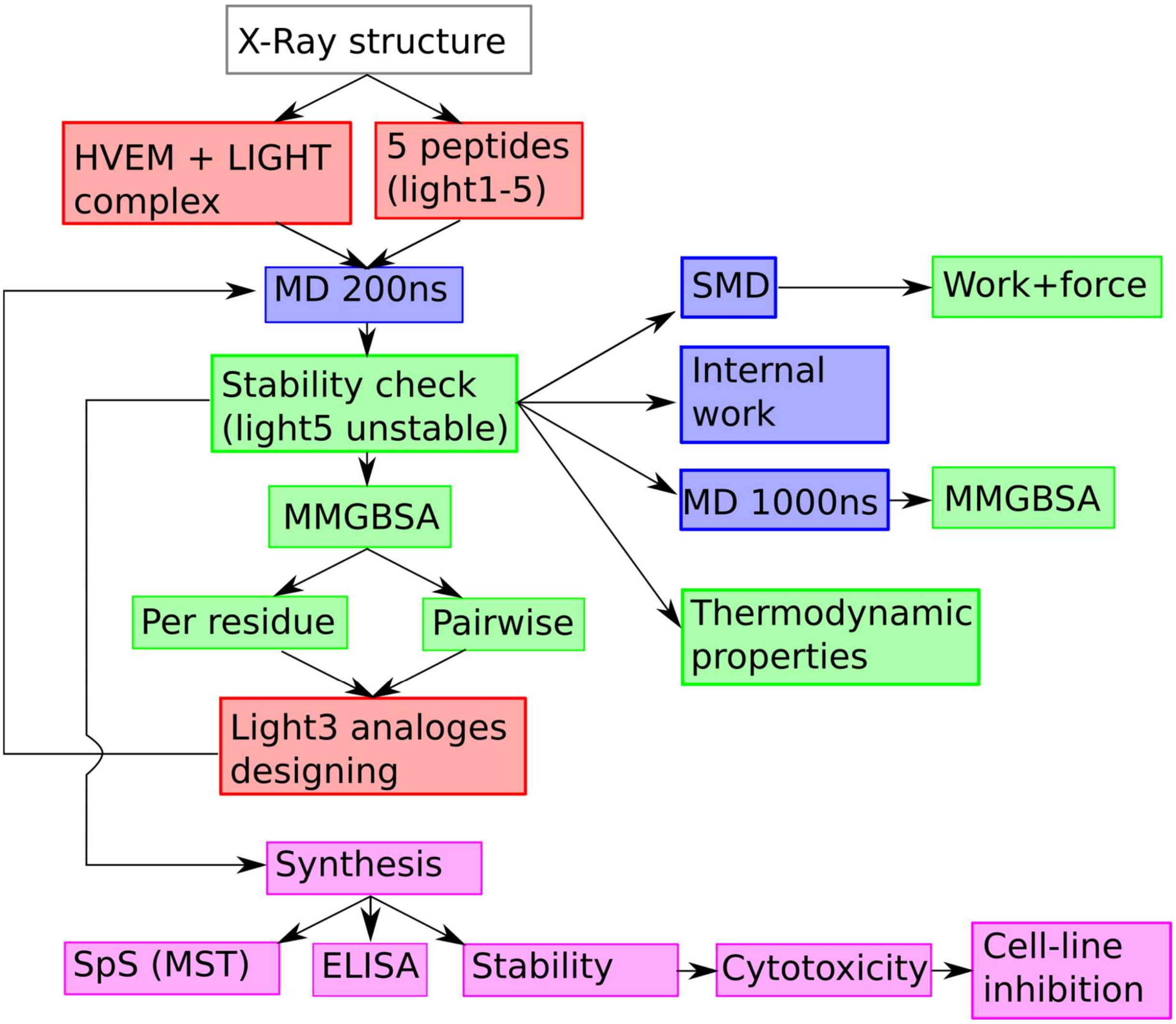

Figure S11. *Schematic overview of the integrated workflow. Processes involving manual analysis are delineated in red, theoretical simulations in blue, theoretical analyses in green, and experimental approaches in purple.*
